# Probiotic supplementation and associated infant gut microbiome and health: a cautionary retrospective clinical comparison

**DOI:** 10.1038/s41598-018-26423-3

**Published:** 2018-05-29

**Authors:** C. Quin, M. Estaki, D. M. Vollman, J. A. Barnett, S. K. Gill, D. L. Gibson

**Affiliations:** Department of Biology, University of British Columbia, Okanagan campus, Kelowna, BC V1V 1V7 Canada

## Abstract

While probiotics are a multi-billion dollar industry, there is little evidence to show that supplementing infants provides any health benefits. We conducted an observational study where 35 of 86 participating mothers self-administered probiotics during breastfeeding, as well as directly to their infants. The primary objective was to determine if probiotic exposure influenced the infants’ fecal microbiome while the secondary objective assessed associated changes to the mothers’ breast milk immunity and infant health. Analysis of infant fecal microbiome throughout the first 6 months of life revealed that probiotics were associated with higher abundances of *Bifidobacterium* at week 1 only. Short-chain fatty acid production and predicted metagenomic functions of the microbial communities were not altered. While probiotics did not alter breast milk immune markers, fecal sIgA responses were higher among probiotic supplemented infants. Surprisingly, this was not associated with better health outcomes, as the probiotic cohort had higher incidences of mucosal-associated illnesses as toddlers. This retrospective clinical comparison suggests that probiotic exposure during infancy has limited effects on gut microbial composition yet is associated with increased infection later in life. These correlative findings caution against probiotic supplementation during infancy until rigorous controlled follow-up studies determining their safety and efficacy have occurred.

## Introduction

In the past few decades microbial research has revealed the importance of the microbiome to human health. While the relationships between specific microbes and health remain largely unknown, epidemiological studies suggest reduced overall diversity of the gut microbiota is associated with diseases like asthma^1^, inflammatory bowel disease^2–4^, and eczema^5^. Microbial ecosystems are established during the first three years of life and form a symbiotic relationship with the host that mutually benefits both. Recent studies have highlighted important roles of gut microbes in regulating various immune events including immune tolerance via modulation of T regulatory (Treg) cells. For example, it has been shown that short-chain fatty acids (SCFA), a by-product of microbial fermentation, can induce the promotion of Treg cell development in the gut^6^. Germ-free mice have reduced Treg cells in the lamina propria compared to mice harbouring a gut microbiota revealing, gut bacteria invoke tolerance to foreign antigens^7^. Microbial metabolites like SCFAs also induce the production of secretory immunoglobulin-A (sIgA)^8^, the main immunoglobulin found in mucus secretions which neutralizes mucosal pathogens^9^. Colonization of the intestinal microbiome during infancy represents a critical time in shaping infant acute and chronic immune-mediated disease susceptibility^10^, and increasing bacterial diversity during this time-period could be an effective preventative strategy to limit adverse immune driven pathologies. Consequently, there has been an increase in consumer interest in probiotic products during the neonatal period as a non-invasive attempt to optimize the infant microbiota. However, there is little evidence to show that probiotics colonize the neonatal gut of healthy, term infants or influence infant health.

The probiotic market has grown into a global, multi-billion dollar industry. The World Health Organization defines probiotics as “live microorganisms, which when administered in adequate amounts confer a health benefit on the host”^11^. This broad definition allows a range of products to be defined as a “probiotic” including foods, infant formula, animal feeds, dietary supplements, and pharmaceutical products. As a result, probiotics encompass a range of regulatory infrastructures with varying requirements for demonstrating safety and efficacy. Not only does this complicate the functional characterization of probiotics, but also the process of manufacturing controls and standards to assure a high-quality product. The consequences of this lack of regulatory oversight are increasingly evident: a premature infant died from gastrointestinal (GI) mucormycosis after being administered a probiotic, manufactured in the USA, which was contaminated with an opportunistic pathogenic mold^12^. Not only does this emphasize that probiotics must be manufactured in accordance with Good Manufacturing Practises, but also highlights the need for expansive probiotic research in vulnerable populations such as infants, prior to health care recommendations and consumer consumption.

Currently, evidence supporting health claims for probiotic products varies widely and the results of observational studies and randomized controlled trials in infants are conflicting. For example, a systematic review concluded that probiotics may be beneficial in preventing eczema in infants^13^. However, another study found probiotics to be a significant risk factor for Vancomycin-resistant Enterococcus colonization and was suggested to mediate the acquisition and transfer of resistance genes of bacteria^14^. Despite this, surveys have shown that clinicians and naturopaths recommend probiotic consumption for patients with a variety of pathologies including diarrhea, ulcerative colitis, and infant colic due to their low cost, over-the-counter availability, and acceptable safety profile in healthy adults^15–18^. The typical probiotic strains manufactured include *Bifidobacterium* and lactic acid bacteria such as *Lactobacillus* species. While these bacteria are present in low abundance in the human intestine, their use dominates the probiotic industry because they are relatively easy to cultivate given their historical use in the dairy industry allowing for large scale production^19,20^. However, there is a lack of evidence that these probiotics influence the gut microbiome when given exogenously and their effect on health outcomes in infants are largely unknown.

Despite the lack of research on the effects of probiotics on infant microbial colonization and health, we unexpectedly found that 40% of women participating in a prospective cohort study focused on fish oil supplementation in the Okanagan Valley (British Columbia, Canada) were self-administering probiotic supplements during lactation, as well as giving probiotics directly to their infants. Therefore, the primary objective of this study was to determine if probiotic exposure during the first 6 months of life, either through maternal supplementation or directly to the infant, correlated with changes in the infant microbiome. The secondary objective was to assess if probiotic exposure correlated with changes to immune markers in breast milk and infant stool, and incidences of mucosal infections over a two-year period. We found that probiotic supplementation did not alter the overall bacterial community’s richness and evenness (alpha diversity), predicted metagenomic functions, or SCFA production. However, limited changes in bacterial community composition (beta diversity) were observed whereby the dominant genera in the probiotics were differentially more abundant in the probiotics cohort when compared to the no probiotics cohort at one week of age. With respect to the secondary outcomes, we found that probiotic supplementation had no effect on the measured immune markers in the breast milk but did associate with significantly higher sIgA measured from infant stool. However, the increased sIgA levels were not correlated with better health outcomes of toddlers, since parent-reported incidences of mucosal infectious diseases revealed higher disease instances in the probiotics group. This was true regardless of mode of delivery (MOD), presences of household pets, siblings, and preschool/daycare attendance as analysed using a multi-model approach. Thus, herein we raise concerns about the safety of probiotics in infants based on retrospective data. These correlative findings should be interpreted cautiously given the unstandardized nature of this study.

## Results

### Baseline characteristics of participants

**Figure 1:**
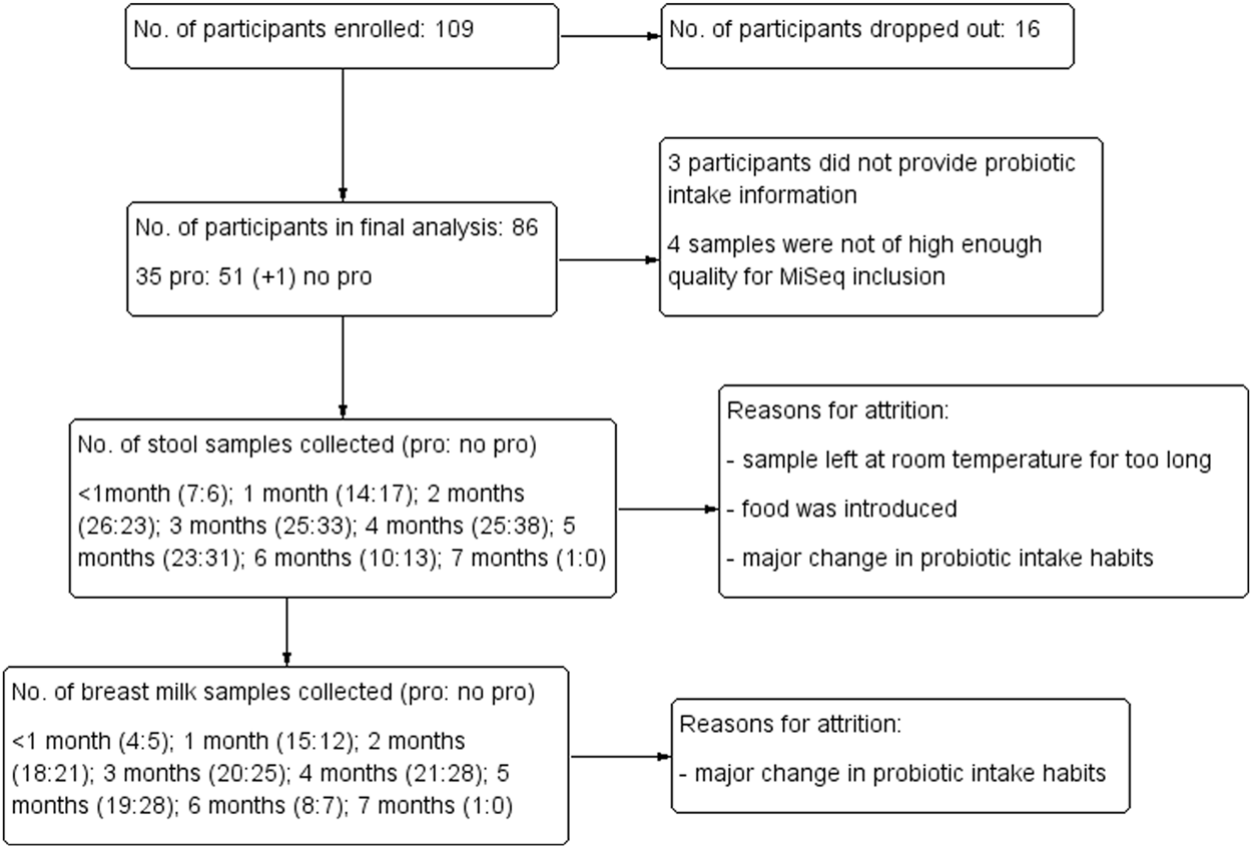
Flow of participants through the trial.

Of the 109 participants enrolled, 86 mother-infant pairs, with an additional infant (1 twin set) were included in the final analysis (Fig. 1). Of these, 40% self-administered probiotics, which are presented in Table 1. In total, 52 infants were not exposed to probiotics and 35 were exposed to probiotics during the first 6 months of life either through their mother’s breast milk (n = 17), directly supplemented (n = 6), or both (n = 12). Subcategorizing participants resulted in 13 “low” probiotic consumers and 22 “high” probiotic consumers. The demographic and clinical characteristics between the participants categorized in the probiotics or no probiotics supplementing groups were comparable (Table 2). There were no differences in maternal age, health, or education between the two groups. Our cohort predominantly included Euro-Canadian women with post-secondary education. Likewise, the clinical characteristics of the infants at birth were similar between the two groups. The infants were born 39.8 ± 1.38 standard deviation (SD) weeks of gestation in the no probiotics group and 39.9 ± 1.67 SD weeks in the probiotics group. The birthweights of the infants in these groups were 7.84 ± 1.06 SD lbs in the supplemented group and 7.72 ± 0.90 SD lbs in the non-supplemented group. Similar in both groups, 71% of infants received their recommended immunizations. Six infants were fed probiotics directly resulting in their samples being categorized as the “probiotics” category whereas the mother’s breast milk was categorized as “no probiotics” in our analysis. Demographic and clinical data was not available for one infant because they were adopted. In addition to demographic and clinical characteristics of the participants we collected environmental and socioeconomic factors (Table 3) which may impact infant health, such as presence of household pets, siblings, and daycare attendance. As the original study was on the effects of fish oil supplementation, we also included fish oil as a potential confounding variable. Of the participants that reported infant illnesses, 63% had pets with a roughly equal split between probiotics (30%) and no probiotics (33%) participants; 77% of the participants had siblings and 48% of these siblings, or the infant themselves, were in daycare or preschool during the study (22% no probiotics: 26% probiotics). Using food frequency questionnaires, it was determined that yogurt consumption was comparable between the two groups and was omitted as a potential confounding variable (Table 3). Environmental and socioeconomic data was missing for two participants (1 probiotic and 1 no probiotic participant) and therefore were excluded in the multi-model analysis. Likewise, a meconium and a seven-month sample were only available from the probiotics group and therefore microbial comparisons were not made at these time points.

**Table 1:** Probiotics consumed by participants.

No.	Brand & Probiotic	Species	Genus	Daily Ave. CFU	Group
*Mother*					
1	Avena Originals, Proteolytic probiotics	*L*. *plantarum*, *L*. *acidophilus*, *L*. *casei*, *B*. *bifidum*, *B*. *longum*	*Lactobacillus* & *Bifidobacterium*	4.03E + 10	High
2	Bio K plus Webber Naturals, Probiotic	*L*. *acidophilus*, *L*. *casei*, *L*. *rhamnosus* *L*. *casei*, *B*. *breve*, *B*. *longum* ssp. *longum*, *L*. *rhamnosus* (HA-111, HA-500), *L*. *acidophilus L*. *plantarum*, *L*. *helveticus*	*Lactobacillus* & *Bifidobacterium*	1.70E + 10	High
3	BioMass, LB17 Live probiotics	*L*. *acidophilus*, *L*. *bulgaricus*, *L*. *casei*, *L*. *fermentum*, *L*. *plantarum*, *L*. *brevis*, *L*. *amylovorus*, *L*. *buchneri*, *L*. *acetotolerans*, *B*. *bifidum*, *B*. *longum*, *P*. *pentosaceus*, *P*. *halophilus*, *P*. *damnosus*, *S*. *thermophilus*, *L*. *lactis*	*Lactobacillus*, *Bifidobacterium*, *Streptococcus* & *Pediococcus*	1.00E + 07	Low
4	Exact, Complete probiotics	*L*. *casei*, *L*. *rhamnosus*, *L*. *acidophilus*, *B*. *longum* ssp. *longum*, *B*. *breve*	*Lactobacillus* & *Bifidobacterium*	2.0E + 10	High
5	Genestra Brands, HMF forte probiotic	*L*. *acidophilus* (CUL 60), *L*. *acidophilus* (CUL 21), *B*. *bifidum*, *B*. *animalis* subsp. *lactis*	*Lactobacillus* & *Bifidobacterium*	2.00E + 10	High
6	Innate Flora 50–14 Natural Factors double strength acidophilus & Bifidus astragalus	*L*. *acidophilus*, *L*. *casei*, *L*. *plantarum*, *L*. *rhamnosus*, *L*. *salivarius*, *L*. *brevis*, *L*. *bulgaricus*, *L*. *gasseri*, *L*. *lactis*, *B*. *longum*, *B*. *bifidum*, *B*. *infantis*, *S*. *thermophilus* *L*. *rhamnosus*, *L*. *acidophilus*, *B*. *bifidum*	*Lactobacillus*, *Bifidobacterium* & *Streptococcus*	6.32E + 09	High
7	Jamieson, Acidophilus	*L*. *acidophilus*, *L*. *rhamnosus*	*Lactobacillus*	1.07E + 09	High
8	Natural Factors, Acidophilus and bifidus double strength	*L*. *rhamnosus*, *L*. *acidophilus*, *B*. *bifidum*	*Lactobacillus* & *Bifidobacterium*	1.00E + 10	High
9	Natural Factors, Acidophilus and bifidus double strength Trophic, Acidophilus Plus	*L*. *rhamnosus*, *L*. *acidophilus*, *B*. *bifidum* *L*. *rhamnosus* R0011, *L*. *casei*, *B*. *longum*, *L*. *acidophilus*, *S*. *thermophilus*, *L*. *bulgaricus*	*Lactobacillus* & *Bifidobacterium*	1.47E + 10	High
10	Natural factors Ultimate multiprobiotic	*L*. *casei*, *L*. *rhamnosus*, *L*. *acidophilus*, *L*. *plantarum*, *L*. *rhamnosus* (B), *B*. *breve*, *B*. *longum*, *B*. *bifidum*, *L*. *lactis*, *L*. *debruceckii* ssp. *bulgaricus*, *L*. *helveticus*, *L*. *salivarius*	*Lactobacillus* & *Bifidobacterium*	1.2E + 10	High
11	Natural world, Vita Flora RT	*L*. *acidophilus*, *L*. *rhamnosus*, *L*. *bulgaricus*, *B*. *longum*, *B*. *bifidum*, *L*. *salivarius*, *L*. *plantarum*, *B*. *lactis*	*Lactobacillus* & *Bifidobacterium*	“rarely”	Low
12	New Roots Herbal, Acidophilus Ultra	*L*. *acidophilus*, *B*. *longum* ssp. *longum*, *B*. *infantis*, *B*. *breve*, *L*. *plantarum*, *L*. *rhamnosus* R0011, *L*. *rhamnosus* R1039, *L*. *helveticus*, *L*. *casei*, *L*. *salivarius* ssp. *S*. *thermophilus*, *L*. *delbrueckii* ssp. *bulgaricus*	*Lactobacillus*, *Bifidobacterium* & *Streptococcus*	2.20E + 09	High
13	Progressive, HCP 30 full spectrum	*L*. *rhamnosus*, *B*. *breve*, *L*. *acidophilus*, *B*. *bifidum*, *B*. *longum*, *L*. *salivarius*	*Lactobacillus* & *Bifidobacterium*	7.50E + 09	High
14	Renew life Florasmart	*L*. *acidophilus*, *B*. *bifidum*, *B*. *longum*, *L*. *casei*, *L*. *rhamnosus*, *L*. *salivarius*	*Lactobacillus* & *Bifidobacterium*	1.50E + 09	High
15	Renew life Ultimate flora	*B*. *animalis* ssp. *lactis*, *L acidophilus*, *B*. *animalis* ssp. *lactis*, *L*. *paracasei*, *B*. *longum*, ssp. *longum*, *L*. *plantarum*, *L*. *salivarius*, *B*. *bifidum*, *B*. *longum* ssp. *Infantis*, *B*. *animalis* ssp. *lactis*, *L*. *casei*, *L*. *rhamnosus*	*Lactobacillus* & *Bifidobacterium*	1.80E + 10	High
16	Unknown Brand*	Unknown species	Unknown genus	1 per 2 days	Low
17	Unknown Brand*	Unknown species	Unknown genus	1 per week	Low
***Mother & Infant***					
1	Flora,Udo’s choice: Super 8 plus probiotic Natural factors, double strength ultimate Natural factors, Ultimate multiprobiotic Renew life, Ultimate flora & BioGaia Probiotics (infant)	*L*. *acidophilus*, *L*. *rhamnosus* HA-111, *L*. *rhamnosus* HA-114, *L*. *plantarum*, *B*. *bifidum*, *L*. *casei*, *B*. *longum*, *L*. *salivarius* *L*. *casei*, *L rhamnosus*, *B*. *breve*, *B*. *longum*, *L*. *acidophilus*, *L*. *plantarum*, *L*. *rhamnosus* (*bifidus*), *B*. *bifidum*, *L*. *fermentum*, *B*. *lactis*, *L*. *salivarius*, *L*. *paracasei* *L*. *casei*, *L*. *rhamnosus*, *L*. *acidophilus*, *L*. *plantarum*, *L*. *rhamnosus* (B), *B*. *breve*, *B*. *longum*, *B*. *bifidum*, *L*. *lactis*, *L*. *debruceckii* ssp. *bulgaricus*, *L*. *helveticus*, *L*. *salivarius* *B*. *animalis* ssp. *lactis*, *L acidophilus*, *B*. *animalis* ssp. *lactis*, *L*. *paracasei*, *B*. *longum*, ssp. *longum*, *L*. *plantarum*, *L*. *salivarius*, *B*. *bifidum*, *B*. *longum* ssp. *Infantis*, *B*. *animalis* ssp. *lactis*, *L*. *casei*, *L*. *rhamnosus* *L*. *reuteri* ssp. *protectis*	*Lactobacillus* & *Bifidobacterium*	7.28E + 10	High
2	Genestra Brands, HMF powder probiotic & BioGaia Probiotics (infant)	*L*. *acidophilus* (CUL 60), *L*. *acidophilus* (CUL 21), *B*. *bifidum*, *B*. *animalis* ssp. *lactis* *L*. *reuteri* ssp. *protectis*	*Lactobacillus* & *Bifidobacterium*	6.8E + 09	High
3	Innate Flora 50–14 Genestra Brands, HMF Natogen (infant)	*L*. *acidophilus*, *L*. *casei*, *L*. *plantarum*, *L*. *rhamnosus*, *L*. *salivarius*, *L*. *brevis*, *L*. *bulgaricus*, *L*. *gasseri*, *L*. *lactis*, *B*. *longum*, *B*. *bifidum*, *B*. *infantis*, *S*. *thermophilus* *L*. *acidophilus* (CUL 60), *L*. *acidophilus* (CUL 21), *L*. *paracasei* (CUL-08), *B*. *animalis* (CUL-62)	*Lactobacillus*, *Bifidobacterium & Streptococcus*	3.45E + 09	High
4	Innovite Health Family Progressive, HCP 30 full spectrum, & BioGaia Probiotics (infant)	*L*. *acidophilus* *L*. *rhamnosus*, *B*. *breve*, *L*. *acidophilus*, *B*. *bifidum*, *B*. *longum*, *L*. *salivarius* *L*. *reuteri* ssp. *protectis*	*Lactobacillus* & *Bifidobacterium*	2.03E + 10	High
5	Jamieson, Acidophilus & Unknown Brand* (infant)	*L*. *acidophilus*, *L*. *rhamnosus* Unknown Brand	*Lactobacillus* & Unknown Genus	“sometimes”	Low
6	Metagenics, Ultraflora balance & BioGaia Probiotics (infant)	*L*. *acidophilus*, *B*. *lactis* *L*. *reuteri* ssp. *protectis*	*Lactobacillus* & *Bifidobacterium*	8.13E + 09	High
7	Metagenic, Ultraflora Acute Care Metagenics, Ultraflora balance Metagenics, Ultraflora immune health Metagenics, Ultraflora immune booster Metagenics, Ultraflora Women’s Thorne Research, Sacro B & BioGaia Probiotics (infant)	*B*. *lactis*, *L*. *rhamnosus*, *S*. *boulardii* *L*. *acidophilus*, *B*. *lactis* *L*. *acidophilus*, *B*. *lactis* *L*. *paracasei*, *L*. *plantarum* *L*. *rhamnosus*, *L*. *reuteri* *S*. *boulardii* *L*. *reuteri* ssp. *protectis*	*Lactobacillus*, *Bifidobacterium* & *Saccharomyces*	1.03E + 10	High
8	Natural Factors double strength acidophilus & Bifidus astragalus & Genestra Brands, HMF Natogen (infant)	*L*. *rhamnosus*, *L*. *acidophilus*, *B*. *bifidum* *L*. *acidophilus* (CUL 60), *L*. *acidophilus* (CUL 21), *L*. *paracasei* (CUL-08), *B*. *animalis* (CUL-62)	*Lactobacillus* & *Bifidobacterium*	6.67E + 09	High
9	Natural factors Ultimate multiprobiotic & BioGaia Probiotics (infant)	*L*. *casei*, *L*. *rhamnosus*, *L*. *acidophilus*, *L*. *plantarum*, *L*. *rhamnosus* (B), *B*. *breve*, *B*. *longum*, *B*. *bifidum*, *L*. *lactis*, *L*. *debruceckii* ssp. *bulgaricus*, *L*. *helveticus*, *L*. *salivarius* *L*. *reuteri* ssp. *protectis*	*Lactobacillus* & *Bifidobacterium*	1.2E + 10	High
10	RepHresh Pro-B probiotic & BioGaia Probiotics (infant)	*L*. *rhamnosus*, *L*. *reuteri* *L*. *reuteri* ssp. *protectis*	*Lactobacillus*	1.73E + 09	High
11	Unknown Brand* & BioGaia Probiotics (infant)	*L*. *acidophilus* *L*. *reuteri* ssp. *protectis*	*Lactobacillus*	4–5 capsules per week	Low
12	Unknown Brand* & BioGaia Probiotics (infant)	Unknown Species *L*. *reuteri* ssp. *protectis*	Unknown Genus & *Lactobacillus*	1.00E + 08	Low
***Infant***					
1	BioGaia Probiotics (infant)	*L*. *reuteri* ssp. *protectis*	*Lactobacillus*	1.00E + 08	Low
2	BioGaia Probiotics (infant)	*L*. *reuteri* ssp. *protectis*	*Lactobacillus*	8.00E + 07	Low
3	BioGaia Probiotics (infant)	*L*. *reuteri* ssp. *protectis*	*Lactobacillus*	1.67E + 07	Low
4	BioGaia Probiotics (infant)	*L*. *reuteri* ssp. *protectis*	*Lactobacillus*	2.90E + 07	Low
5	BioGaia Probiotics (infant)	*L*. *reuteri* ssp. *protectis*	*Lactobacillus*	2.50E + 07	Low
6	Flora, Udo’s choice: super toddler’s probiotics (infant)	*L*. *casei*, *B*. *bifidum*, *S*. *thermophilus*, *L*. *bulgaricus*, *L*. *acidophilus*, *B*. *breve*, *B*. *infantis*	*Lactobacillus*, *Bifidobacterium & Streptococcus*	8.50E + 08	Low

Abbreviations: Participant number (No.), Colony forming unit (CFU).

^*^Average daily CFU consumption could not be calculated.

**Table 2:** Baseline Demographic, and Clinical Characteristics.

Total Subjects	Probiotics	No Probiotics	Significance
Gender (n, male: female)	17:18	27:25	NS
Caesarian deliveries (%)	35.2	29.4	NS
Maternal age (yr)	32.3 ± 3.5	31.5 ± 4.0	NS
Weight at birth (g)	3557 ± 481	3500 ± 410	NS
Length at birth (cm)	52.45 ± 3.21	51.50 ± 3.75	NS
Vaccinated (%)	71.4	71.2	NS

Data is presented ± SD. NS denotes not significantly different.

**Table 3:** Environmental and Socioeconomic Variables.

Total Subjects	Probiotics	No Probiotics	Significance
Siblings (%)	37.0	40.7	NS
Preschool/daycare (%)	25.9	22.2	NS
Fish oil exposure (%)	29.6	33.3	NS
Household pets (%)	33.3	29.6	NS
Yogurt consumption (%)	71.4	70.6	NS

NS denotes not significantly different.

### Overall microbial richness and evenness are not different between the probiotics and no probiotics infant groups

**Figure 2:**
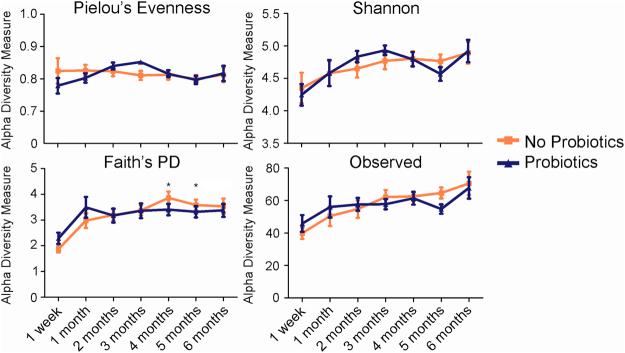
Alpha diversity measures of fecal microbiota over a 6-month period of probiotics and no probiotics exposed infants. The alpha diversity in the infants’ stool did not differ between the two groups at any time point (1 week to 6 months of age). While the two groups were not different from each other, Faith’s phylogenetic diversity in the no probiotics group significantly increased at months 4 and 5 when compared to the first week, whereas the probiotics group showed no significant increases. *Denotes *P* < 0.05.

To understand if probiotic exposure during the first 6 months of life influence the infant microbial ecosystem, we examined their fecal microbiota using 16S rRNA target gene high-throughput sequencing. Alpha diversity, a measure of species richness and evenness within a single sample, was determined using the following indices: Pielou’s Evenness, Shannon’s diversity index, Faith’s phylogenetic diversity (PD), and observed species richness. Pielou’s evenness measures community evenness whereas Shannon’s diversity index is a quantitative measure of community richness. Faith’s PD similarly measures community richness but also incorporates phylogenetic relationships between features. The observed species index, which measures the number of unique features observed within a single sample, was chosen as a proxy to assess the exposure route (direct infant supplementation or via breast milk) using a two-way ANOVA. No difference was detected between the two routes and data was combined for all presented microbial analyses (Table 4). None of the alpha diversity measures were significantly different between the probiotics and no probiotics group at any age; however, increases in microbial richness appeared to occur at different time-points (Fig. 2) in infants not supplemented with probiotics. While there were no changes in Pielou’s evenness, Shannon’s diversity, or observed species richness from one week to 6 months of age in either cohort, Faiths PD showed a significant increase in community richness in the no probiotics group at months 4 (mean = 3.9; 95% confidence interval (CI)[3.4, 4.4], *P* < 0.05), and 5 (mean = 3.6; 95% CI [3.2, 4.0], *P* < 0.05) when compared to the first week of life (mean = 1.9; 95% CI [1.5, 2.2], *P* < 0.05), whereas infants exposed to probiotics did not. Subcategorizing infants into high and low probiotic exposure does not change the results of Faith’s PD, but showed additional increases in observed species richness at months 5 and 6 in the no probiotics group when compared to the first one week of life (Figure S1.A). Overall, these results reveal that infants who are exposed to probiotics have comparable, or lower, microbial diversity to non-supplemented infants.

**Table 4:** Two factor ANOVA on exposure route using observed species richness.

Age	Factor	No Probiotics	Probiotics	95% CI	Sig
1 week	Directly	43.67	41.50	[−17.19, 12.86]	NS
	Lactation	37.88	51.20	[−0.93, 27.58]	
1 month	Directly	47.67	64.71	[−6.341, 40.44]	NS
	Lactation	48.42	56.42	[−12.08, 28.07]	
2 months	Directly	54.16	59.08	[−11.74, 21.58]	NS
	Lactation	54.67	56.23	[−12.84, 15.96]	
3 months	Directly	58.74	62.67	[−11.39, 19.25]	NS
	Lactation	61.11	56.60	[−17.56, 8.55]	
4 months	Directly	60.94	61.08	[−12.84, 13.11]	NS
	Lactation	61.20	60.52	[−11.83, 10.49]	
5 months	Directly	59.76	56.50	[−16.22, 9.70]	NS
	Lactation	61.47	54.63	[−18.15, 4.47]	
6 months	Directly	66.88	74.33	[−17.72, 32.62]	NS
	Lactation	69.64	67.56	[−24.73, 20.56]	

NS denotes not significantly different. No probiotics and probiotics are expressed as means.

### Probiotic supplementation is associated with higher Bifidobacterium at one week of age

**Figure 3:**
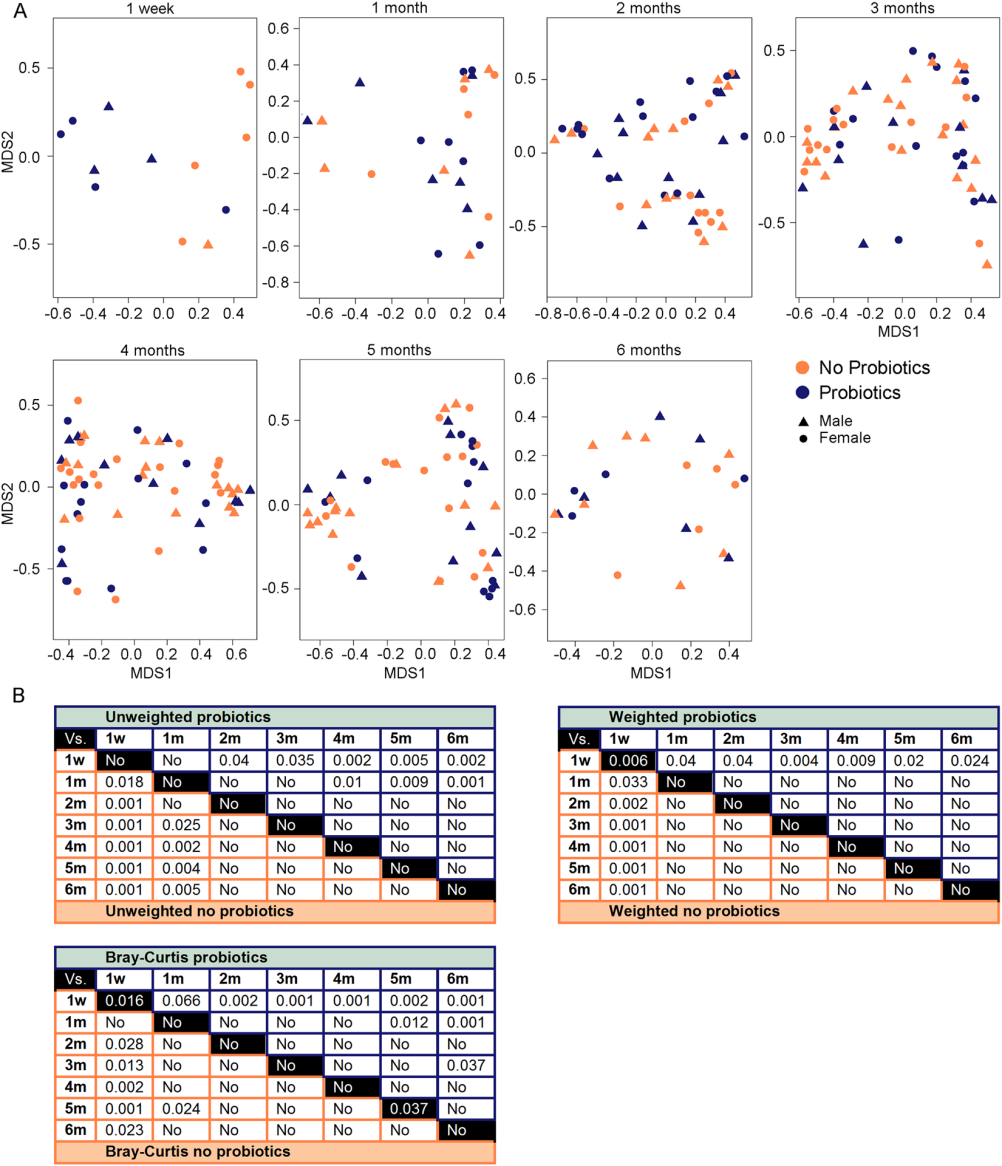
Beta diversity assessment of microbial communities over a 6-month period of probiotics and no probiotics exposed infants. (**A**) PCoA plots based on the Bray-Curtis dissimilarity distance show no distinct clustering of microbial communities at any age except for at 1 week. (**B**) Table summary of PERMANOVA results ran on the Bray-Curtis dissimilarity, and weighted and unweighted UniFrac distances between the two groups. The values at the intersect of a blue cell (probiotics group) and an orange cell (no probiotics group) show the estimated *P* value of the corresponding time points.

Several beta diversity measures were used to determine the extent of change in microbial community composition between the groups. Principal coordinate analysis (PCoA), an ordination method which utilizes a distance matrix constructed from non-Euclidean distance measures, was first used to visualize Bray-Curtis dissimilarity between samples (Fig. 3A). This visualization revealed no distinct clustering between the probiotics and no probiotics group at any time point except at one week of age. To further determine, through statistical testing, whether bacterial populations were influenced by probiotic exposure, a permutational multivariate analysis of variance (PERMANOVA) was applied to the Bray-Curtis dissimilarity matrix. The results of the PERMANOVA agreed with the visualization and showed no main difference between the two groups at any age except for at one week of age (*P* = 0.016) and 5 months (*P* = 0.037). In addition to the Bray-Curtis dissimilarity matrix, weighted and unweighted UniFrac measures were used to assess phylogenetic distances between sets of taxa^21^. The unweighted UniFrac is a qualitative measure based on the presence or absence of bacteria whereas the weighted UniFrac considers the relative abundance of the taxa. In this analysis, the unweighted UniFrac showed that the overall composition of the samples’ community in the first 6 months of life were similar across groups (Fig. 3B). As expected, the microbial presence changed over the 6-month period in both the probiotics and no probiotics group; however, these changes were comparable between both groups. Differences in relative abundances of taxa (weighted UniFrac) between the probiotics and no probiotics group were detected at one week of age (*P* = 0.006) (Fig. 3B). While the clustering did not change (Figure S1B), these results did not persist when subdivided into high and low probiotic exposure (Table S1), likely due to the low sample size in each group (none n = 6; low n = 3; high n = 4).

**Figure 4:**
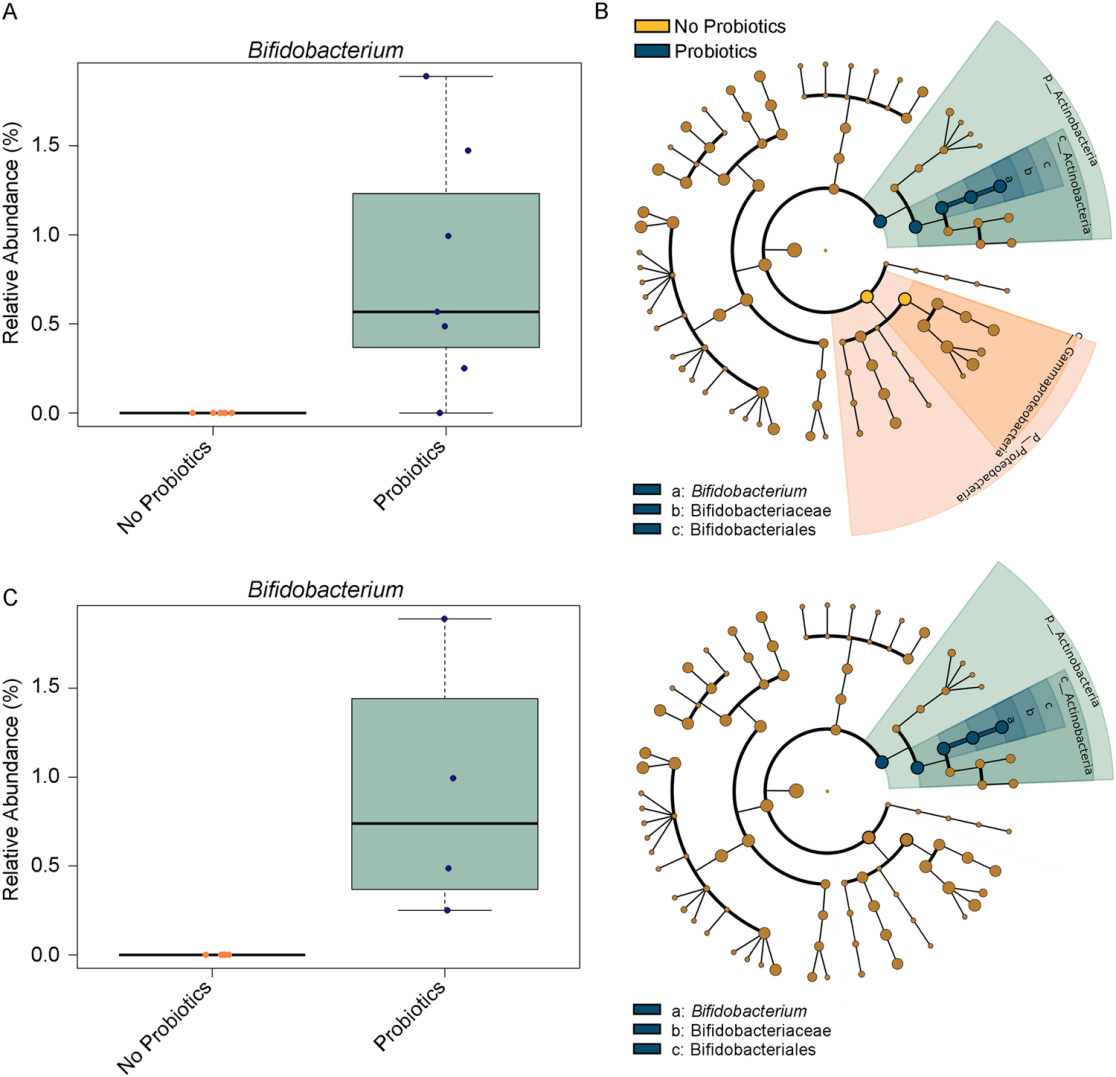
Differences in the abundance of taxa between the groups were assessed using two complimentary approaches at each time-point. (**A**) Differences in the relative abundance of *Bifidobacterium spp*. in the probiotics group at one week of age was detected as significantly different using ANCOM (*P* < 0.05, F-statistic = 7.5). (**B**) LEfSe results showing significantly different taxa between fecal samples between probiotics and no probiotics infants at one week of age. The cladogram reports the taxa showing different abundance values according to LEfSe. Colors indicate the lineages that are encoded within corresponding samples. Higher taxa abundance in the probiotics group is colored blue whereas higher taxa abundance in the no probiotics group is colored orange. (**C**) Infants directly supplemented with BioGaia according to the manufacturers recommendations show similar results to (**A** and **B**). *P* < 0.05.

To determine which taxa were differentially abundant between the two groups, analysis of composition of microbiomes (ANCOM) was performed at each age^22^. Overall, ANCOM did not identify significant features at any age with the exception of *Bifidobacterium spp*. being more abundant in the probiotics group at one week of age (Fig. 4A). A linear discriminant analysis of effect size (LEfSe)^23^ validated these results (Fig. 4B) and with the 8 significantly discriminative features identified before internal Wilcoxon testing, showed a significant (α = 0.05) increase in the abundance of *Bifidobacterium longum* in the probiotics group as well as an increase in Gammaproteobacteria in the no probiotics group at 1 week of age. The cladogram shows the bacterial distribution in the sample groups and differences in abundances between them, according to LEfSe, were displayed as colors and circle’s diameters. LEfSe identified several other taxonomic differences between the probiotics and no probiotics groups at other ages (Figure S2). However, as the ANCOM results did not find any differences at these ages, we cautiously excluded these results to avoid false positive discovery, though future studies may benefit in using these results as possible *a priori* genera of interest. These results did not change when the infants directly consuming BioGaia according to the manufacturers recommendations were analyzed separately (4 BioGaia supplemented infants: 6 non-supplemented infants), in an attempt to standardize the brand and dose of probiotics consumed (Fig. 4C). Overall, there is evidence that the community composition and differential taxon abundances are associated with probiotic supplementation in infants at one week of age using the whole cohort (unstandardized) and a small subset of infants consuming the same brand of probiotic.

### Probiotics are not associated with gut microbiome function

**Figure 5:**
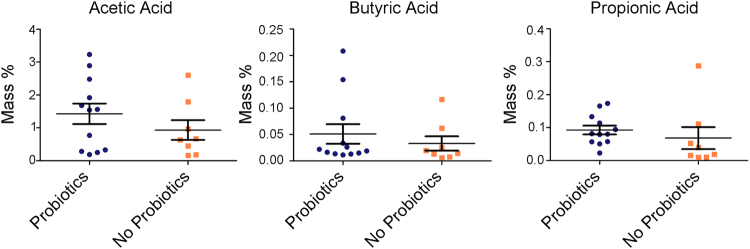
Abundances of fecal SCFAs acetic acid, butyric acid, and propionic acid between probiotics and no probiotics exposed infants at 5 months of age expressed as mass % (g SCFA/g dry weight stool).

The trillions of bacteria present in the gut have redundant genetic contribution and therefore similar functions. Predicted metagenomic functions of the microbial communities using phylogenetic investigation of communities by reconstruction of unobserved states (PICRUSt)^24^, revealed no predicted functional differences between the two cohorts. Another method of assessing the functions of the intestinal microbial communities is through measuring fecal SCFAs, the by products of bacterial fermentation of carbohydrates. We analyzed the presence of fecal acetate, butyrate, and propionate which have been found to play an important role in regulating intestinal homeostatic and immune responses^25^. Probiotic exposure was not associated with changes in the abundances of these SCFAs found in the infants’ stool showing that despite some taxonomic differences, neither predicted gut microbial function nor SCFAs were different between groups (Fig. 5).

### Probiotic supplementation is not associated with breast milk immunity

**Figure 6:**
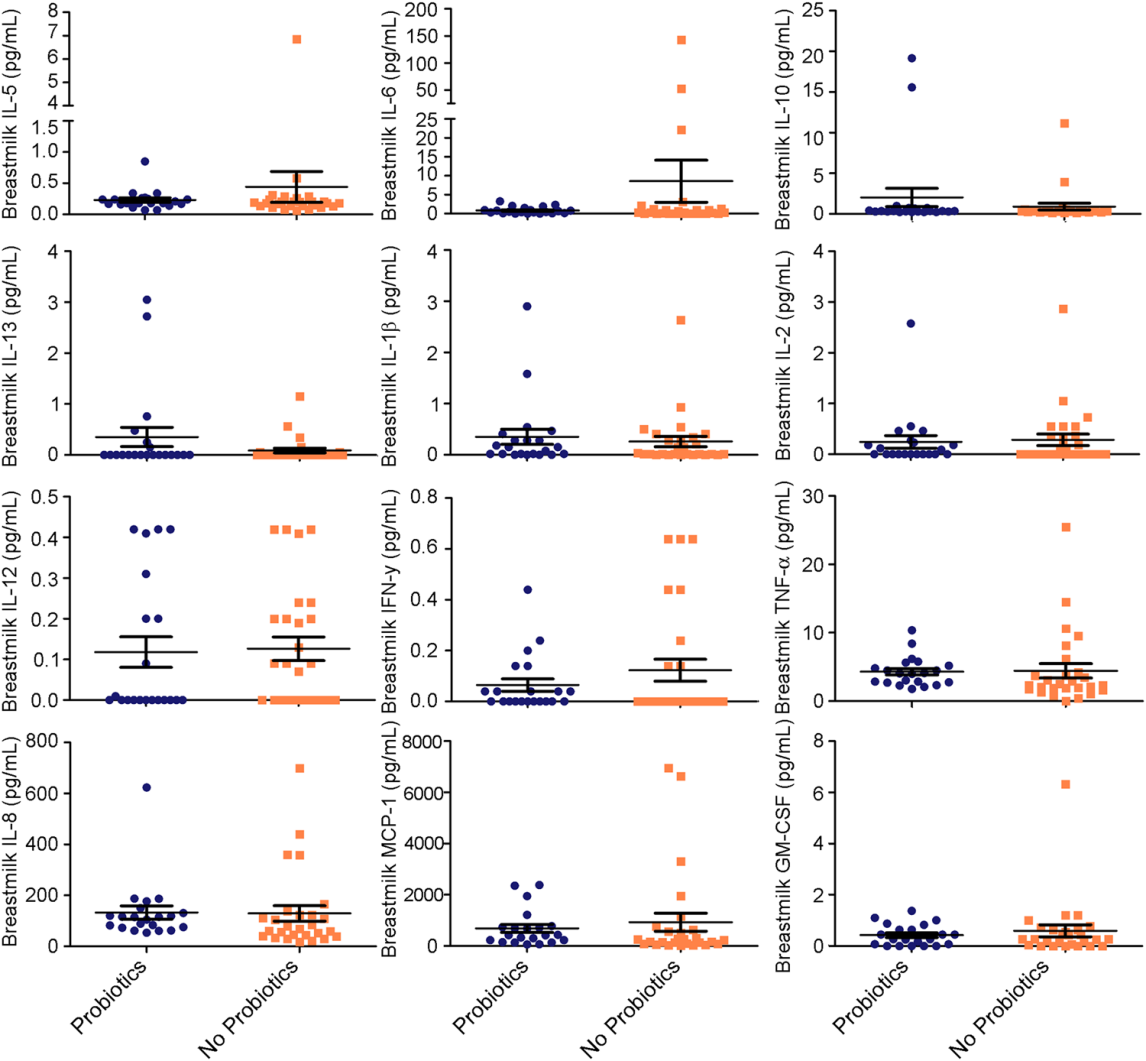
Immune markers in breast milk of mothers with or without probiotic supplementation at 5 months. The scatter dot plot shows the mean and standard error mean.

**Figure 7:**
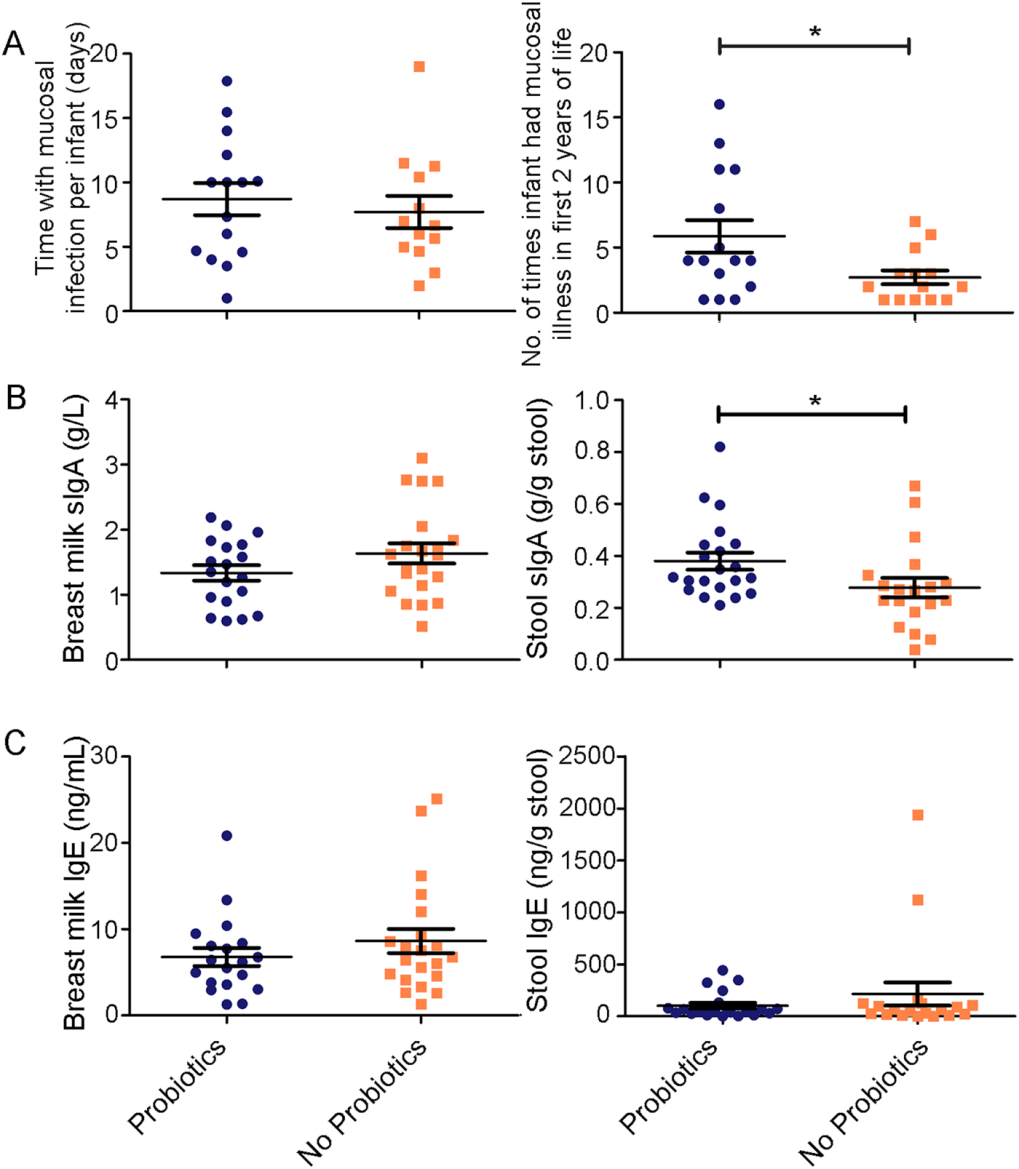
Infant exposure to probiotics correlates with increased mucosal illness during the first 2 years of life and corresponding immune responses at 5 months of age. (**A**) Infants exposed to probiotics had significantly higher reported mucosal infections compared to the no probiotics group but were able to clear infections at similar rates. (**B**) Probiotic exposure during the first 6 months is associated with no changes in breast milk sIgA but modest increases in fecal sIgA. (**C**) There were no differences in either breast milk or infant stool IgE levels. *Denotes *P* < 0.05.

Innate and adaptive immune cells found in breast milk protect infants from mucosal infections while their immune system develops^26^. To determine if probiotic supplementation influenced the protective potential of breast milk, immune cytokines were examined in the breast milk. In addition, IgE was analyzed to determine if probiotics were associated with markers of allergies whereas sIgA was analyzed as a marker of mucosal protection against enteric disease^27^. The multivariate generalized linear models (GLM) of the breast milk cytokines showed no differences between groups (likelihood ratio test = 23.8, adj. *P* = 0.5) (Fig. 6). Similarly, there were no significant differences between levels of sIgA or IgE in breast milk (Fig. 7), showing that probiotic supplementation was not associated with protective immune responses in breast milk.

### Higher probiotic intake correlates with increased mucosal infections

To determine if probiotics are correlated with infant mucosal health including enteric and respiratory infections, sickness incidence reports were collected over a two-year period. The responses from these reports are presented in Table S2. Oral, respiratory, and gastrointestinal infections were classified as mucosal infections and included in subsequent analysis. There was a 43% (n = 15) response in participants that were self-administering with probiotics and a 27% (n = 14) response in the participants who did not self-administer probiotics. We found that both cohorts were able to clear infections at comparable rates (probiotics: 9.16 ± 1.21; no probiotics: 7.71 ± 1.25); however, infants exposed to probiotics during the study had a higher frequency of mucosal infections reported (5.87 ± 1.23) in the first 2 years of life compared to the no probiotics group (2.71 ± 0.53) (Fig. 7A). Results from a multi-model inference approach confirmed these findings while accounting for covariates. Of the 64 possible models, 5 models within two AICc (corrected Akaike information criteria) of the best model were averaged. The explanatory variables MOD and presence of pets did not appear in any of the top model sets, while siblings and fish oil supplements appeared in 2, preschool in 1, and probiotics appeared in all five sets, highlighting the importance of this variable (Table 5). Model averaging indicated that probiotic supplementation had the strongest, positive effect on the incidences of sickness (estimated coefficient (EC) 3.3, *P* = 0.025, EC/standard error >2) while presence of a sibling (EC 2.61, *P* = 0.114), fish oil supplementation (EC −2.15, *P* = 0.150), and preschool attendance (EC 1.19, *P* = 0.429) did not have a significant effect and had higher variability (EC/standard error <2).

**Table 5:** Results of multi-model inference on probiotics supplementation and incidences of mucosal infection.

	Estimated coefficient	Adjusted SE	P	Relative variable importance	n-containing models (n/5)
Intercept	2.363	1.768	0.18		
Probiotics-yes	3.262	1.454	**0**.025*	1.0	5
Siblings-yes	2.612	1.653	0.115	0.47	2
DHA-yes	−2.150	1.493	0.150	0.41	2
Preschool-yes	1.186	1.498	0.429	0.09	1

As probiotics have been shown to influence the humoral immune responses, we examined sIgA and IgE in the infants’ stool to determine if sickness incidence corresponded with humoral immune markers. Despite probiotics having no effect on the immunoglobulins in the mothers’ breast milk, probiotics were associated with a modest increased sIgA levels measured in infants’ stool at 5 months of age (difference between means = 0.10 ± 0.05; 95% CI [0.001, 0.20], *P* < 0.05) (Fig. 7B). Similar to the mothers’ breast milk, there was no significant difference in the IgE measured from the infants’ stool (difference between means = −113.1 ± 114.8; 95% CI [−352.6, 126.5], *P* = 0.16) (Fig. 7C) suggesting no relation with allergy susceptibly.

## Discussion

Probiotics have been proposed to influence a wide range of health outcomes presumably by altering the intestinal microbiota and consequently immunity^28^ but research is limited on the clinical effects of probiotic exposure during infancy. Despite the lack of supporting evidence, we unexpectedly found that 40% of women participating in a cohort study in the Okanagan Valley were self-administering probiotic supplements while breastfeeding their infants. Although this study was not designed to examine the effects of probiotics, the limited evidence supporting a practise that is becoming popular merits a post-hoc analysis of the collected data.

In Western populations, there has been a documented step-wise progression from infancy towards an adult-like microbiome. Early colonizers in breastfed infants include Firmicutes, *Staphylococcus*, *Streptococcus*, and Enterobacteraceae. These are followed by Enterococcus and *Lactobacillus* and then finally *Bifidobacterium* and *Clostridium*^29^. The bacterial communities present in the breast milk have been suspected of being transferred from the mothers’ intestine via an entero-mammary pathway mediated by the immune system^30,31^. Research reports the mechanism involved in this transfer involves bacteria in the lumen of the mothers intestine being captured by dendritic cells through openings in the tight junctions of the intestinal epithelium and transporting the bacteria to the mammary gland via the lymphatic system^32^. Therefore, it has been suggested that alterations to the mother’s intestinal microbiota through probiotics may change the colonization patterns in their infants and resulting immunity.

In this retrospective clinical comparison, infants exposed to probiotics had increased *Bifidobacterium* in their stool at one week of age. Previous studies have shown that after one week of age, *Bifidobacterium* becomes detectable in infant faeces^33^ suggesting that probiotics may promote slightly earlier colonization of *Bifidobacterium*. Congruent with these findings, an increase in *Bifidobacterium* at one week is typically accompanied by a decrease in Enterobacteriaceae^33^, a type of Gammaproteobacteria. The results from the LEfSe analysis showed significantly higher Gammaproteobacteria in the non-supplemented cohort at one week of age, though these findings were not validated by ANCOM. These differences could be attributed to differences in which these methods test the data. Aside from the differences in relative taxa abundance at one week of age, this study found no differences in community richness based on several alpha diversity measures, or overall community composition differences based on the unweighted UniFrac results, at any tested age. Additionally, the taxon abundances were similar between the two groups at 1 month of age and remained similar up to 6 months of age. This agrees with previous literature which showed no effect of probiotics taken from 36 weeks gestation to 3 months post-parturition on offspring diversity at 3 months or two years^34^. Here, we also show that despite differences in taxon abundances at one week of age, the predicted functions of the microbiome did not differ between the two groups at any age, nor did the measured SCFAs at five months, suggesting a potentially short period following birth where probiotics may exert an effect. Still, minor perturbations to the establishment of the microbiome may yet have health consequences.

Intestinal colonization by the microbiome during infancy corresponds to the development of immune tolerance and disease susceptibility, and host-microbial interactions in early life influence life-long function of the immune system^35^. While the exact intricacies of this relationship are still unknown, an increase in *Bifidobacterium* in breast fed infants has been associated with improved health in adults^36^ and has been shown to increase the production of IgA^37^. To examine the effects of probiotics on infant mucosal immunity, we non-invasively measured immunogenic markers and tracked the frequency of infant enteric and respiratory infections. However, counter to our expectations given the increase in *Bifidobacterium*, we found that probiotic exposure in infants during the first 6 months of life correlated with increased incidences of mucosal infections over a 2-year period. This immunologic effect was irrespective of the immunogenic components of the mothers’ breast milk which showed no difference in inflammatory cytokines or humoral markers. The probiotic infant cohort did, however, have significantly increased sIgA in their stool at 5 months which agrees with other recent findings. For example, a recent study of Italian newborns showed that fecal sIgA values were higher in those whose mothers took high-dose probiotics than the controls, despite a lack of differences in mothers’ breast milk sIgA levels^38^. However, similar to this study it is difficult to disentangle whether the increased sIgA is beneficial to the infant, or a result of increased mucosal infections during this period of time. We hypothesize that the altered microbial community at one week of age may disturb the ‘normal’ development of the infant immune system. Germ-free models have shown that in the absence of microbes, the immune system is underdeveloped (reviewed here^35^) and that inflammation induced by commensal microbes acts to ‘mature’ the immune system while simultaneously protecting the host from pathogenic organisms. However, not all bacteria induce inflammatory responses. For example, Gram-negative commensal organism *Bacteroides thetaiotaomicron* induces the production of RegIIIγ by Paneth cells, but Gram-positive *Bifidobacterium longum* does not^39,40^. It is tempting to speculate that the equilibrium between inflammation and regulation in the gut is disrupted by probiotic supplementation resulting in decreased intestinal inflammation at one week of age and subsequent immune maturation. In contrast to our findings that probiotics are associated with an increase in mucosal infections, a recent study in India has shown that an oral administration of ~10^9^ *Lactobacillus plantarum* ATCC strain 202195 combined with prebiotics, like fructooligosaccharide, in the first few days of life reduced the incidence of sepsis^41^. This symbiotic preparation has been shown to colonize infants for up to 4 months when administered to neonates in the first week of life^42^. In our study, *Lactobacillus plantarum* strains were consumed by 12 of the 35 probiotic participants; however, there was no evidence that *Lactobacillus* spp. were different between the infants who were exposed to probiotics and those who were not at any time point. This could be due to different doses, loss of probiotic viability which has been shown to drastically decrease by 10 weeks, the absence of the prebiotic, or inherent differences in the microbiome of westernized babies compared to Indian babies. It is also tempting to speculate that compared to Canadian babies, Indian babies are exposed to more pathogenic bacteria which would induce early inflammation. In this case, ‘anti-inflammatory’ bacteria may help to balance the inflammatory responses.

Overall, this study shows that probiotics rich in *Bifidobacterium*, *Lactobacillus*, and *Streptococcus* are associated with increased *Bifidobacterium* in infants at one week of age, increased sIgA in stool at 5 months, and increased mucosal infections reported in the first 2 years of life compared to infants not exposed to probiotics. This correlative evidence suggests that early probiotic exposure may influence immune development through altered taxa abundances at one week of age and alarmingly may increase susceptibility to mucosal infections; however, these findings should be interpreted with cation as this study has several limitations. This observational study was not initially designed to examine the effects of probiotics. As such, participants consumed varying brands and doses of probiotics, although arguably all participants consumed the same genus of probiotics including *Lactobacillus*, *Bifidobacteria* and/or *Streptococcus*. In an effort to standardize the brand and dose of probiotics consumed by the baby directly, the sample size was reduced considerably generating an additional limitation. While 16S amplicon sequencing has less detection power than the whole genome shotgun approach, studies suggest that 16S can assign genera with high confidence >90% of the time^43^, which would allow this study to detect significant increases in probiotic genera in the infant gut. Another significant limitation of our study was that the response rates on the sickness incidence forms were low (probiotics n = 15; no probiotics n = 14). Despite follow-up phone calls and emails every 3 months, relying on care-giver observations of illness posed a challenge which was only partially mitigated by the comprehensive questionnaire. Moreover, the power calculation accounted for differences in the microbiome, our primary objective. This study was not designed for an epidemiological study on health. Given these limitations, causal relationships between probiotics and infant gut health cannot be made from this study, and the results should be interpreted cautiously. However, given that limited changes to the microbiome were observed in our retrospective data, and potential negative health outcomes were associated with probiotic supplementation during infancy, health care providers and parents should use probiotics cautiously until adequately sized, prospective controlled clinical trials are performed.

This study shows that the prevalence of use of probiotics among Canadians, during lactation, is high. Studies in Canada, South Australia and Kuwait all agree that the primary reasons for using natural health products like probiotics is to maintain and promote health and to build the immune system^44,45^. A common perception among consumers is that probiotics are safe and are not associated with risks. However, human and animal studies show that probiotics may enhance tumorigenesis in colorectal cancer^46^, increase risk of mortality in severe acute pancreatitis^47^, and transfer virulence factors to pathogenic or opportunistic pathogens^14^. Yet despite high utilization rates and potential harmful effects, physicians do not routinely question their patients about probiotic usage^48–50^ and consumers do not disclose natural health product usage unless directly asked^48^. One proposed reason for this is that healthcare practitioners, nutritionists, dieticians, pediatricians and consumers all have a positive attitude towards natural health products^50^ and do not consider them to be a “drug”. While in actuality, the safety and efficacy of probiotics depends upon the amount and dosage, the characteristics of the consumer, and the context that they are used^51^. As of yet there is not enough evidence-based science to recommend probiotic supplements for infants. This study is intended to promote awareness of the risks probiotics may pose to infants and to encourage follow-up, controlled studies prior to issuing recommendations to mothers.

## Material and Methods

### Power analysis

A power analysis was computed using G*Power for the original cohort study^52,53^. Based on another study analyzing infant microbiota^54^, the effect size of 0.13 was chosen. A sample size of 25 per group was calculated to be sufficient to detect microbial differences in beta diversity analysis using a Type I error of 0.05 and a power of 0.9. To account for loss of subjects through the study and imprecise knowledge on the overall error we selected a minimum sample size of 30 to ensure that the power will be at least 90%.

### Study design

Our research protocol and methods were approved by the UBC Clinical Research Ethics Board and BC Interior Health Ethics Board and written informed consent was obtained from each participant at enrolment. All methods were performed in accordance with the UBC Clinical Research Ethics Board and BC Interior Health Ethics Board guidelines and regulations. This paper is reported following the ‘strengthening the reporting of observational studies in epidemiology’ (STROBE) cohort study checklist^55^. Women were recruited through pamphlets and posters at family clinics, fitness centres, publicly funded healthcare providers (Interior Health centres), gyms, crises centers, and child daycares to capture a representative population of the Okanagan Valley. Recruitment pamphlets were also included in hospital discharge packages. Participation included healthy, full-term infants who were predominantly breastfed by healthy mothers from birth to the introduction of solid foods. Women-infant pairs were excluded if the infant had low-birth weight or was diagnosed with congenital defects. We collected information about delivery (mode, place, and caregiver), antibiotic treatment, anthropometric measurements (body weight, length, and head circumference), formula intake, vaccination compliance, and demographics of the parents. Recruitment for the trial began January 2014 and ended September 2015 resulting in participation from 109 healthy, lactating women in the Okanagan valley. Of these, 86 were included in the final analysis with 35 women in the probiotics group and 51 women, plus an additional twin, in the no probiotics group. The participants self-administering probiotics were further categorized as “low” probiotic consumers if they consumed on average less than 1 billion colony forming units (CFU) per day for the duration of the study, were inconsistent with probiotic intake, or CFU per day could not be calculated. Participants were categorized as “high” probiotic consumers if they consumed on average greater than or equal to 1 billion CFU per day on average for the duration of the study. Probiotic supplementation and dietary intake was tracked throughout the study using a validated 24-hour recall questionnaire adapted from the Alberta Pregnancy Outcomes and Nutrition (APrON) cohort study^56^. We tracked illnesses from reported occurrence and duration of enteric and respiratory illness symptoms by caregivers. Studies have shown that self-reporting morbidity data using a recall method can introduce error attributed to memory loss^57,58^. As such, we provided each participant with a link to an online sickness incidence form which they could fill out immediately at first signs of infection. This questionnaire was outlined in a previous study^59^. Briefly, the questionnaire queried the occurrence and duration of illness symptoms and whether the diagnosis was made personally or by a medical professional. It included 22 listed symptoms such as cough, phlegm, diarrhea, vomiting, fever, etc., and space to fill out additional symptoms. Our focus was on mucosal infections which included respiratory and Gastrointestinal (GI; bacterial and viral) illnesses. Additionally, email reminders were sent every 3 months. As the two cohorts shared similar baseline characteristics, response rates were unlikely to be biased towards either group.

### Sample collection

We collected the stool from the infants at approximately 1 week of age (8.6 ± 0.95 days of age in the no probiotics group and 8.3 ± 1.3 days of age in the probiotics group), and then again monthly until 6 months of age. Concurrently, we collected breast milk samples from the mothers for comparisons. Instructions and collection packages containing a sterile spatula to collect stool and sterile tubes for breast milk samples were given to the participants monthly. Parents were instructed to store the stool and breast milk samples in their home freezer until we collected them on dry ice within 3 days post-collection. Samples were subsequently homogenized and stored as described before^60^. Briefly, infant stool was homogenized with a pestle and mortar while kept frozen using liquid nitrogen prior to DNA extraction. Samples were excluded if the infant was exposed to antibiotics within 2 weeks of the sample collection date.

### High throughput sequencing

#### Sequencing methods

Total DNA was extracted from fecal samples using the QIAamp DNA stool mini kit (Qiagen; Cat No 51504), according to manufacturer’s specifications. 5 ng of extracted DNA, normalized using a Nanodrop 1000 spectrophotometer, was used in a PCR reaction to amplify the V3-V4 region of the 16S ribosomal DNA using 341F and 805R primers^61^ attached to the Illumina adapter overhang. A second PCR using the Nextera XT dual index kit^62^ attached unique identifiers to both 5′ and 3′ ends. Agencourt Ampure XP beads (Beckman Coulter) were used to clean all PCR products. Pooled amplicons were checked for quality and quantity on the Experion Automated Electrophoresis System (Bio-Rad) and 2 × 300 bp, paired end reads were sequenced on a MiSeq system at The Applied Genomics Core (TAGC) in Edmonton, AB.

#### Bioinformatics

All bioinformatics processing was performed within QIIME2^63^ using the various built-in wrappers cited below. Paired-end sequences from two MiSeq runs underwent separate quality-filtering, dereplication, chimera removal, denoising, and merging using the DADA2^64^ plugin with default settings. An alternative to standard OTU clustering, DADA2 produces an amplicon sequence variant (ASV) table that is a higher-resolution analogue of traditional OTU tables with fewer spurious sequences. The two resulting ASV tables were then merged prior to subsequent analyses. A Naïve Bayes classifier was trained on the specific region targeted by our primer sets using the most recent available version of the Greengenes (13_8) sequences. Taxonomic classification was collapsed and assigned at the genus level. For microbiome analyses encompassing phylogenetic information, MAFFT-aligned^65^ sequences were used to produce a phylogeny tree using FastTree2^66^ with default settings. All used software packages, versions, and parameters are available under the “provenance” section of the QIIME2 feature-table artifact available at: https://osf.io/pj7fh/. This file can be viewed locally on a browser by drag and dropping the file onto https://view.qiime2.org/. PICRUSt^24^ analysis was conducted on 97% similarity clustered OTUs as picked using VSEARCH^67^ in QIIME version 1.9.0 as PICRUSt requires closed-reference OTU picking using the Greengenes database. OTUs were normalized by copy number and a new matrix of predicted functional categories were created using the Kyoto encyclopedia of Genes and Genomes (KEGG) database. The statistical analysis of metagenomic profiles (STAMP)^68^ software package was used to analyze the predicted metagenomic function of the communities. At each age, the two groups were compared using White’s non-parametric t-test and confidence intervals were calculated using a difference between mean proportions percentile bootstrapping method. A Benjamini-Hochberg false discovery rate was used to correct for multiple testing. The predicted functional table for PICRUSt analysis is available on the Open Science Framework platform.

#### Microbial Analysis

Microbial data was rarefied at a sampling depth of 4196. This resulted in a loss of 5 samples (4 no probiotic samples at months 1, 4, 5, 5; 1 probiotic sample at month 2). To reduce noise in the multivariate analyses, features with fewer than 10 counts across all samples or appearing in fewer than 5 samples were removed. Comparison of taxa diversity between infant fecal samples was determined using the following alpha diversity measures: Pielou’s evenness, Shannon’s diversity index, Faith’s PD, and observed species richness. To visualize broad trends of overall bacterial community populations between the two groups, a PCoA was used on a Bray-Curtis dissimilarity matrix with the *Vegan* package in R version 3.3.0. A PERMANOVA (α = 0.05) with 999 random permutations was run on the Bray-Curtis dissimilarity matrix as well as with the weighted and unweighted UniFrac distance matrices^21^ to statistically determine differences between groups. Microbial differences were further explored using LEfSe^23^, which identifies biologically relevant features between two or more biological conditions, and ANCOM^22^, which utilizes pairwise log ratios between all features followed by a Mann-Whitney *U* test in determining differentially abundant taxa between two biological conditions. For the LEfSe analysis the groups were treated as classes and a multiclass comparison was performed on the features using the Kruskal-Wallis to detect main effect differences followed by Wilcoxon rank-sum test for pairwise comparisons, with a *P* value cut-off of 0.05 and a linear discriminant analysis (LDA) score of 2.0 for identifying discriminative features. The differentially abundant features were then visualized in a cladogram. If results differed between the two methods, we proceeded with the findings that were cross validated by both LEfSe and ANCOM methods. This process was repeated using only the infants directly supplemented with BioGaia following manufacturers recommendations, to understand the effects of a more standardized probiotic supplementation at one week of age.

### Short-chain fatty acid analysis

Intraluminal SCFA (acetate, butyrate, and propionate) were extracted from 20 randomly selected samples using isopropyl alcohol from 5-month old infants’ fecal contents as described previously^69^. The amount of acetic, propionic, and butyric acid were analyzed from fecal samples via direct-injection gas chromatography as previously described^70^, and expressed as mass % (g of SCFA per g of dry weight stool).

### Cytokine analysis

sIgA, IgE, and the expression of pro- and anti-inflammatory cytokines (colony-stimulator granulocyte-macrophage colony-stimulating factor (GM-CSF), interferon gamma (IFN-γ), Interleukin (IL)-1β, IL-2, IL-6, IL-8, IL-10, IL-12 (p70), monocyte chemoattractant protein 1 (MCP-1), tumor necrosis factor alpha (TNF-α), IL-13, and IL-5) were measured from whole breast milk and infant stool supernatant at 5 months of age, serviced by Eve Technologies (evetechnologies.com; Calgary, Canada). To prepare stool supernatant, infant stool was diluted in a 1:4 w/v ratio of phosphate-buffered saline, homogenized at 10 °C, and then centrifuged at 4 °C to collect the supernatant.

### Statistical analysis

All results are expressed as mean values ± standard errors of the mean unless otherwise stated. For cytokine analysis, a multivariate approach was used to simultaneously assess differences in abundances of each cytokine between the probiotics groups (Yes/No) while accounting for possible interactions between cytokines. We implemented multiple GLMs fit with a negative binomial link function using the *mvabund*^71^ package (version 3.13.1) in R (version 3.4.1)^72^ and assessed the regression assumptions visually using the mean-variance diagnostic plot. The model *P* value was calculated using default settings with 999 resampling iterations using the PIT-trap resampling method which accounts for correlation in multiple testing. For sIgA, IgE, infants age difference at one week, and SCFAs analyzed on 5-month data, Students T-tests were used. A two-way ANOVA was used to assess the impact of both exposure routes (directly or through breast milk) at each age using the observed species richness to determine if routes could be pooled together. Given that fecal samples from each subject were not completely matched at each time point, repeated measures ANOVAs could not be conducted. As such, each age was tested separately for microbial analysis. For comparisons between alpha diversity measures, the Mann-Whitney *U* test was used when data was deemed not normally distributed as determined by a Shapiro-Wilk normality test. Analyses were performed using GraphPad Prism 5 software, and a *P* value < 0.05 was considered statistically significant.

### Multi-model inference

To examine the relationship between probiotics supplementation and incidences of infant mucosal-related infections within the first 2 years of life we utilized a multi-model approach within an Akaike information criteria (AIC) and model averaging framework^73^. All multi-model analyses were performed using R statistical program using the *MuMIn*^74^ package (1.40.4). First, a full global model was built with the number of reported incidences as the response variable and the following covariables: siblings (yes/no), preschool attendance (yes/no), presence of house-hold pets (yes/no), mode of delivery (vaginal/c-section), fish oil supplementation by mother (yes/no), and probiotics supplementation (yes/no). We used binary categorization of probiotics supplementation instead of their dose-designated categories (none/low/high) as the uneven sample size across these levels limited the information provided to the models. However, exploratory analysis of this category revealed that the incidences of sickness between low and high groups were very similar and therefore pooling them is unlikely to affect the outcome of the models. Model fit was visually inspected using diagnostic plots of the residuals. All 64 combinations of subset models (Table S3) using the variables within the global model were performed using the *dredge* function which ranks the sub-models based on their AICc, a derivative of AIC that penalizes models with low sample sizes. As no single model yielded high explanatory power, model averaging was performed on a subset of models within 2 AICc of the best model and cross-validated on another subset including all models within the 95% cumulative AICc weights. As both approaches yielded similar results, here we report the former’s averaged estimated coefficients, adjusted standard errors which accounts for model selection uncertainty, individual variable *P* values, and their relative importance factor.

### Availability of Data and Clinical Trial Registry Platform

The data set supporting the results of this article is available in the QIITA database repository, StudyID: 11380 at http://qiita.microbio.me and the Open Science Framework platform https://osf.io/pj7fh/.

The clinical trial is registered at https://clinicaltrials.gov (2017/09/28) NCT03297801.

## Electronic supplementary material

Supplemental Information
