# Supplementary material for: Probiotic supplementation and associated infant gut microbiome and health: a cautionary retrospective clinical comparison
Source: Sci Rep. 2018 May 29;8:8283. doi: 10.1038/s41598-018-26423-3 (PMC5974413; doi:10.1038/s41598-018-26423-3)
Supplement: Supplementary file 1 — Supplemental Information [file 41598_2018_26423_MOESM1_ESM.doc]

**Probiotic supplementation and associated infant gut microbiome and health: a cautionary retrospective clinical comparison**

Quin C, Estaki M, Pasquale DM, Barnett JA, Gill SK & Gibson DL*

Department of Biology, University of British Columbia,

Okanagan campus, Kelowna, BC, V1V 1V7, Canada

*; corresponding author

Dr. Deanna L. Gibson, Ph.D.

Department of Biology,

University of British Columbia, Okanagan campus

ASC 386, 3187 University Way

Kelowna, BC, V1V 1V7

Canada.

Telephone: (1-250) 807-8790

Email: [deanna.gibson@ubc.ca](mailto:deanna.gibson@ubc.ca)

### **Supplementary Methods:**

### Bioinformatics

For this analysis Deblur was utilized instead of DADA2. Briefly, demultiplexed forward reads were quality filtered based on PHRED scores[^34^](#_ENREF_31) prior to the application of Deblur at a trim length of 160, to maintain a minimum quality score of 20. Samples were rarefied to a sampling depth of 4380 for diversity analysis, which allowed us to retain all our samples. The most recent version of Greengenes (13_8) was used to train a Naïve Bayes classifier with a 99% similarity requirement. Analyzing data using Deblur did not change the previous probiotic results, nor did probiotic level.

**Supplementary Results:**

Observed species richness, Shannon’s diversity index, Pielou’s evenness, and Faith’s phylogenetic diversity (PD) were used to re-assess alpha diversity measures when the probiotic cohort was separated into low and high probiotic exposure. Like the previous analysis, Faith’s PD showed a significant increase in community richness in the non-supplementing group at months 5 (mean = 6.32; 95% confidence interval (CI) [5.7, 7.0], *P* <0.05), and 6 (mean = 6.6; 95% CI [5.6, 7.7], *P* <0.05) when compared to the first week of life (mean = 2.8; 95% CI [1.5, 4.2], *P* <0.05), whereas infants exposed to probiotics did not (Figure S1). Similarily, the observed species richness, showed a significant increase in diversity in the no probiotics group in months 5 (mean = 85.2; 95% CI [75.5, 95.0], *P* <0.05), and 6 (mean = 91.9; 95% CI [74.5, 109.2], *P* <0.05) compared to the first week of life (mean = 43.7; 95% CI [27.9, 59.4], *P* <0.05), whereas observed diversity in the probiotics group did not significantly increase over a six-month period. There was no significant increase in the probiotic groups (low and high) at any time point, nor was there a significant difference between the three groups.

Beta diversity measurements were not changed by separating groups into none, low, and high. Beta diversity was assessed using the following: weighted UniFrac distances, unweighted UniFrac Distances, and Bray-Curtis dissimilarity distance. Figure S1 B shows an ordination PCoA plot of the Bray-Curtis dissimilarity distances. Table summary of the PERMANOVA for the Bray-Curtis shows no significant difference between the noprobiotics and high probiotics group (Table S1.A), and the no probiotics and low probiotics group (Table S1.B).

**Supplementary Discussion:** Overall, this data shows that the probiotics consumed at either a high or low dose does not influence microbial community richness (alpha diversity), but similarly show clustering at one week of age based on Bray-Curtis dissimilarity matrices. While the PERMANOVA does not show a significant effect of low or high supplementation, the *n* values are quite low and may not have enough power for statistical inference.


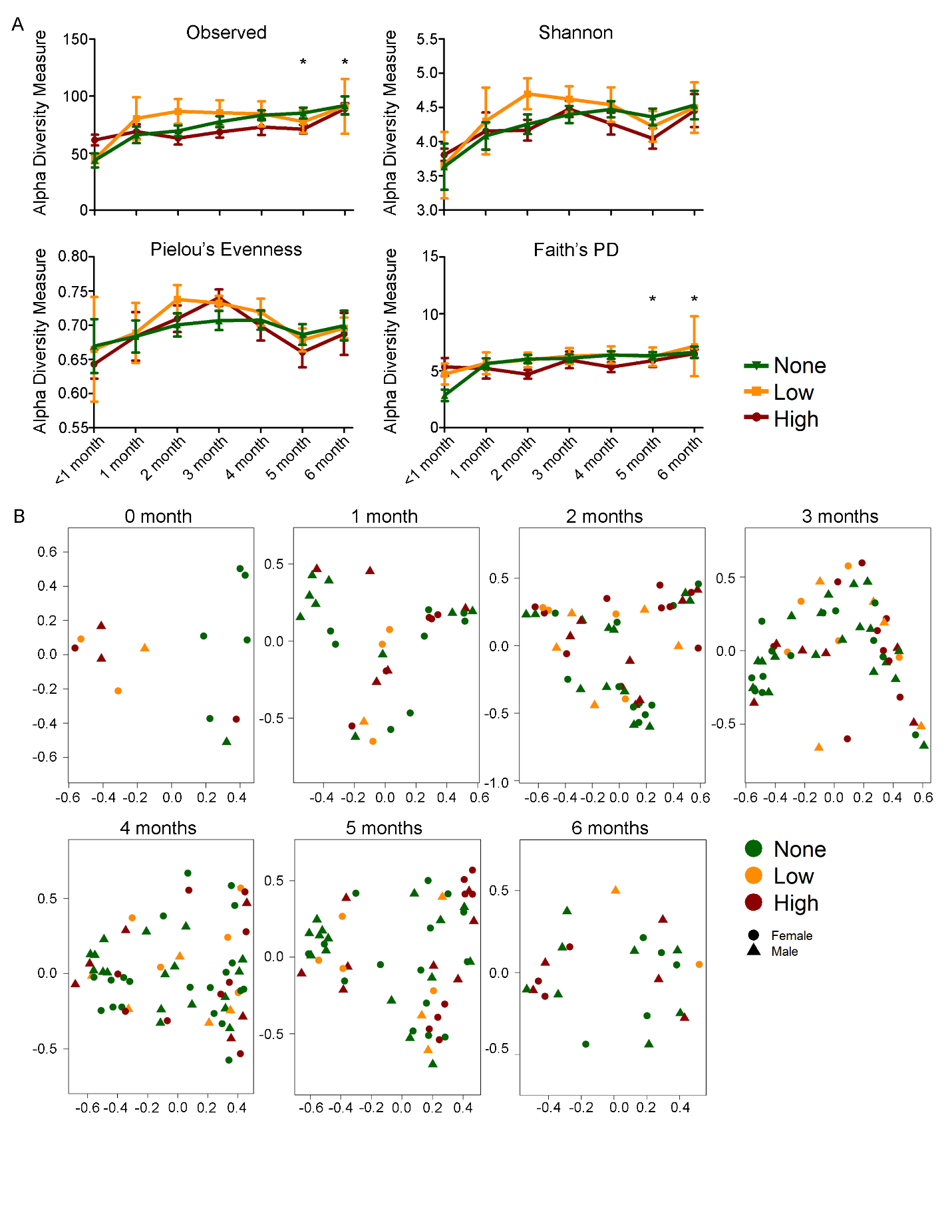


**Figure S1.** Subgrouping the probiotic infants into “high” probiotic exposure and “low” probiotic exposure did not reveal differences in microbial diversity. A) Alpha diversity included observed species richness, Shannon’s diversity index, Pielou’s evenness, and Faith’s phylogenetic diversity. While the three groups were not different from each other, alpha diversity significantly increased in the non-probiotic group at months 5 and 6 compared to one week of age. B) PCoA plots based on Bray-Curtis dissimilarity distances, depicting no significant clustering between the high, low, and no probiotics groups at any age except for one week of age.


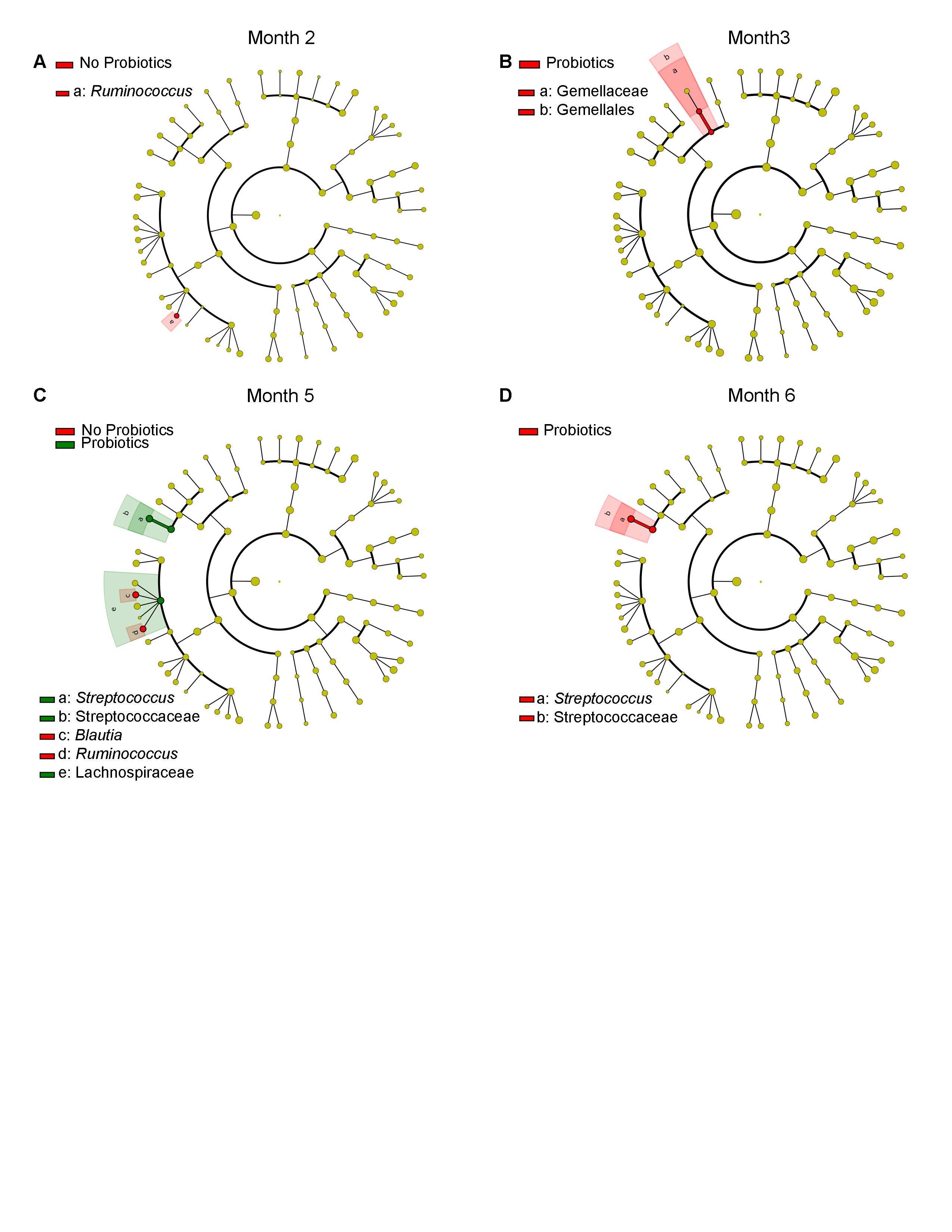


**Figure S2.** Significantly discriminative features identified by LEfSe at months 2, 3, 5 and 6 between the probiotic and non-probiotic infants. The cladogram reports the taxa showing different abundance values according to LEfSe. Colors indicate the lineages that are encoded within corresponding samples. A. *Ruminococcus* was significantly more abundant in the non-probiotic group at 2 months. B. The order Gemellales was more abundant in the probiotic group at 3 months. C. *Streptococcus* and Lachnospiraceae were more abundant in the probiotic group at 5 months whereas the non-probiotic group had significantly more *Blautia* and *Ruminococcus*. D. At 6 months, the probiotic group had a higher abundance of *Streptococcus*. Months 1 and 4 had no significantly dissimilative features between the two groups and are not represented.

**Table S1. PERMANOVA results from Bray-Curtis Dissimilarity measures comparing no probiotic exposure to (A) high and (B) low probiotic exposure**

| A. | High Probiotics | | | | | Bray-Curtis | | | B. | Low Probiotics | | | | | Bray-Curtis | | |
| --- | --- | --- | --- | --- | --- | --- | --- | --- | --- | --- | --- | --- | --- | --- | --- | --- | --- |
|  | Vs | 0m | 1m | 2m | 3m | 4m | 5m | 6m |  | Vs | 0m | 1m | 2m | 3m | 4m | 5m | 6m |
|  | 0m | No | No | No | No | 0.04 | No | 0.04 |  | 0m | No | No | No | No | No | No | No |
|  | 1m | No | No | No | No | No | No | 0.05 |  | 1m | No | No | No | No | No | No | No |
|  | 2m | No | No | No | No | No | No | No |  | 2m | No | No | No | No | No | No | No |
|  | 3m | 0.05 | No | No | No | No | No | No |  | 3m | No | No | No | No | No | No | No |
|  | 4m | 0.03 | No | No | No | No | No | No |  | 4m | No | No | No | No | No | No | No |
|  | 5m | 0.03 | No | No | No | No | No | No |  | 5m | No | No | No | No | No | No | No |
|  | 6m | No | No | No | No | No | No | No |  | 6m | No | No | No | No | No | No | No |
|  | No Probiotics | | | | | | | |  | No Probiotics | | | | | | | |

| **Table S2. Sickness Reports** | | | | | | | | | | | | | | | | | | | | | | | |
| --- | --- | --- | --- | --- | --- | --- | --- | --- | --- | --- | --- | --- | --- | --- | --- | --- | --- | --- | --- | --- | --- | --- | --- |
| Probiotics | Age  (m) | Report | Symptoms | | | | | | | | | | | | | | | | | | Diagnosis | No. Mucosal  Infections  (muc/resp/gastro) | Ave. Duration  (days) |
|  |  |  | 1 | 2 | 3 | 4 | 5 | 6 | 7 | 8 | 9 | 10 | 11 | 12 | 13 | 14 | 15 | 16 | 17 | Other |  |  |  |
| No | 17 | 1 | X |  | X | X |  |  |  |  |  |  |  |  |  |  | X |  | X |  | Cold; resp | 1 | 8 |
| No | 13 | 1 | X |  | X | X | X |  |  |  |  |  |  |  |  |  |  |  |  |  | Cold; resp | 3 | 11.5 |
|  | 14 | 2 | X | X | X | X | X | X |  |  | X |  |  |  |  |  | X | X |  |  | Flu; resp |  |  |
|  | 20 | 3 | X |  | X | X | X | X |  |  |  |  |  |  |  |  |  |  |  |  | Cold; resp |  |  |
| No | 8 | 1 |  |  | X | X |  | X |  |  |  |  |  |  |  |  |  |  |  | lethargic | Cold; resp | 3 | 6.66 |
|  | 12 | 2 | X | X | X |  |  | X |  |  |  |  |  |  |  |  |  |  |  |  | Cold; resp |  |  |
|  | 17 | 3 | X | X | X |  | X |  |  |  |  |  |  |  |  |  | X |  |  |  | Cold; resp |  |  |
| No | 11 | 1 |  |  |  |  | X |  |  |  |  |  |  | X |  |  |  |  |  | Ear pain | Ear infection* | 0 | NA |
| No | 4 | 1 | X |  | X |  | X |  |  |  |  |  |  |  |  |  |  |  |  |  | Cold; resp | 2 | 6 |
|  | 9 | 2 | X | X |  |  |  |  |  |  |  |  |  |  |  |  |  |  |  |  | Cold; resp |  |  |
| No | 0 | 1 |  |  |  |  |  |  |  |  | X |  |  |  |  |  |  |  |  |  | Fever | 0 | NA |
| No | 2 | 1 | X |  | X | X |  |  |  |  |  |  |  |  |  |  |  |  |  |  | Cold; resp | 1 | NA |
| No | 3 | 1 |  |  |  |  |  |  |  |  | X |  |  |  |  |  |  |  |  |  | Fever | 3 | 4.66 |
|  | 14 | 2 | X |  |  | X |  |  |  |  |  |  |  |  |  |  |  |  |  |  | Cold; resp |  |  |
|  | 15 | 3 | X |  |  |  |  |  |  |  | X |  |  |  |  |  |  |  | X |  | Cold; resp |  |  |
|  | 15 | 4 | X |  |  |  |  |  |  |  | X |  |  |  |  | X |  |  |  | Gagging | Cold; resp |  |  |
| No | 15 | 1 |  |  |  |  | X |  |  |  |  |  |  | X |  |  |  |  |  | Reduced appetite | Eczema/allergy | 0 | NA |
|  | 18 | 2 |  |  |  |  |  |  |  |  |  |  |  |  |  |  |  |  |  |  | ear infection* |  |  |
| No | 3 | 1 | X | X | X | X |  | X | X |  |  |  |  |  |  |  |  |  |  |  | Cold; resp* | 5 | 11.25 |
|  | 3 | 2 | X | X | X | X |  | X | X |  |  |  |  |  |  |  |  |  |  |  | Cold; resp* |  |  |
|  | 12 | 3 |  |  |  | X |  |  |  |  |  |  |  |  |  |  |  |  |  |  | Cold; resp |  |  |
|  | 17 | 4 |  |  |  | X |  |  |  |  | X |  |  |  |  |  |  |  |  |  | Cold; resp |  |  |
|  | 18 | 5 |  |  |  |  |  |  |  |  | X |  |  |  |  |  |  |  |  |  | Vaccination reaction |  |  |
|  | 23 | 6 | X |  | X | X |  |  |  |  | X |  |  |  |  |  |  |  | X |  | Flu; resp |  |  |
| No | 4 | 1 |  | X | X |  | X |  |  |  | X |  |  |  |  |  |  |  |  |  | Cold; resp | 6 | 5.66 |
|  | 6 | 2 | X |  | X |  | X | X |  |  |  |  |  |  |  |  |  |  |  |  | Cold; resp |  |  |
|  | 7 | 3 |  |  |  |  |  |  |  |  | X |  | X |  |  |  |  |  |  |  | Gastro |  |  |
|  | 9 | 4 | X |  |  | X |  | X |  |  |  |  |  |  |  |  |  |  |  |  | Cold; resp |  |  |
|  | 11 | 5 |  |  |  |  |  |  |  |  | X |  | X |  |  |  |  |  |  |  | Gastro |  |  |
|  | 11 | 6 |  |  |  |  |  |  |  |  | X |  | X |  |  |  |  |  |  |  | Gastro |  |  |
|  | 15 | 7 | X |  |  |  |  |  |  |  |  |  |  |  |  |  |  |  |  |  | Ear infection* |  |  |
|  | 16 | 8 | X |  |  | X |  |  |  |  | X |  |  |  |  |  |  |  |  |  | Ear infection* |  |  |
| No | 9 | 1 | X |  |  | X |  | X |  |  | X |  |  |  |  |  |  |  |  |  | Cold; resp* | 1 | 3 |
|  | 9 | 2 |  |  |  |  |  |  |  |  |  |  |  |  | X |  |  |  |  |  | Dermal rash |  |  |
| No | 19 | 1 | X |  | X | X | X |  |  | X |  |  |  |  |  |  |  |  |  |  | Cold; resp | 2 | 5 |
|  | 19 | 2 | X |  |  |  |  |  |  | X | X | X |  | X |  | X | X |  | X |  | Croup; resp* |  |  |
| No | 6 | 1 | X | X | X | X | X |  |  |  | X | X |  |  | X |  |  |  |  |  | Gastro | 2 | 7 |
|  | 16 | 2 | X |  | X | X | X |  |  |  | X |  |  |  |  |  |  |  |  |  | Cold; resp |  |  |
| No | 7 | 1 | X |  | X | X | X | X |  |  |  |  |  |  |  | X |  |  |  |  | Cold; resp | 7 | 10.42 |
|  | 8 | 2 | X |  | X | X | X | X | X |  |  |  |  |  |  | X | X |  |  |  | Cold; resp |  |  |
|  | 11 | 3 | X | X | X | X | X | X |  |  |  |  |  |  |  |  | X |  |  |  | Cold; resp |  |  |
|  | 9 | 4 |  |  |  |  |  |  |  |  | X |  |  | X |  |  |  |  |  |  | Flu; resp* |  |  |
|  | 12 | 5 |  |  |  |  |  |  |  |  | X |  |  | X |  |  |  |  |  |  | Flu; resp* and rash |  |  |
|  | 12 | 6 | X |  | X | X | X |  |  |  | X | X |  |  |  |  |  |  |  |  | Gastro |  |  |
|  | 13 | 7 | X |  | X | X | X | X |  |  | X | X |  |  |  |  |  |  |  |  | Gastro |  |  |
|  | 18 | 8 |  |  |  |  |  |  |  |  | X |  |  |  |  |  |  |  |  | Irritable | Fever |  |  |
| No | 16 | 1 | X | X | X | X | X |  |  |  |  |  |  |  |  |  |  |  |  |  | Cold; resp | 1 | 19 |
| No | 4 | 1 |  |  | X | X |  |  |  |  |  |  |  |  |  |  |  |  |  | sneezing | Cold; resp | 1 | 2 |
| Yes | 2 | 1 | X |  | X | X | X |  |  |  |  |  |  |  |  |  |  |  |  |  | Cold; resp | 11 | 10.09 |
|  | 7 | 2 | X | X |  |  |  |  | X |  |  |  |  |  |  |  |  |  |  |  | Cold; resp* |  |  |
|  | 8 | 3 | X |  | X | X |  |  |  |  |  |  |  |  |  |  |  |  |  |  | Cold; resp |  |  |
|  | 8 | 4 | X |  | X | X |  |  |  |  |  |  |  |  |  |  |  |  |  |  | Cold; resp |  |  |
|  | 9 | 5 |  |  |  |  |  |  |  |  |  |  |  |  |  |  |  |  |  | Oozing eye | pink eye |  |  |
|  | 10 | 6 | X | X |  | X | X |  |  |  |  |  |  |  |  |  |  |  |  |  | Cold; resp |  |  |
|  | 15 | 7 |  |  |  |  |  |  |  |  |  |  |  |  |  |  |  |  |  | Reduced appetite | Teething |  |  |
|  | 16 | 8 |  |  |  | X | X |  |  |  |  |  |  |  |  |  |  |  |  |  | Cold; resp |  |  |
|  | 16 | 9 |  |  |  |  |  |  |  |  |  | X |  |  |  |  |  |  |  |  | Gastro |  |  |
|  | 17 | 10 | X |  | X | X |  |  |  |  |  |  |  |  |  |  |  |  |  | Hoarse voice | Cold; resp |  |  |
|  | 19 | 11 | X |  | X | X |  |  |  |  |  |  |  |  |  |  |  |  |  | Hoarse voice | Cold; resp |  |  |
|  | 20 | 12 |  |  |  | X |  |  |  |  |  |  | X |  |  |  |  |  |  | irritable | Gastro |  |  |
|  | 21 | 13 | X |  | X | X |  |  |  |  |  |  |  |  |  |  |  |  |  |  | Cold; resp |  |  |
| Yes | 11 | 1 |  |  | X | X | X | X |  |  |  |  |  |  |  |  |  |  |  |  | Cold; resp | 1 | 10 |
| Yes | 0 | 1 |  |  |  |  |  |  |  |  |  |  |  |  |  |  |  |  |  | Mucous cysts in mouth | Cysts in the mouth; Muc* | 4 | 17.88 |
|  | 0 | 2 |  |  |  |  |  |  |  |  |  | X |  |  |  |  |  |  |  | Pain when placed on back | Reflux* |  |  |
|  | 1 | 3 |  |  |  |  |  |  |  |  |  |  |  |  |  |  |  |  |  | White buildup in mouth | Thrush* |  |  |
|  | 3 | 4 | X |  |  |  | X |  |  |  | X |  |  |  |  |  |  |  |  | Pink eye | Cold; resp & Pink Eye* |  |  |
|  | 11 | 5 |  |  |  |  |  |  |  |  | X |  |  | X |  |  |  |  |  |  | Roseola; resp* |  |  |
| Yes | 2 | 1 |  |  | X | X |  | X |  |  |  |  |  |  |  |  |  |  |  |  | Cold; resp | 13 | 15.46 |
|  | 4 | 2 | X |  | X | X | X | X |  |  |  |  |  |  |  |  |  |  |  |  | Cold; resp |  |  |
|  | 5 | 3 | X | X | X | X | X | X |  |  |  |  |  |  |  |  |  |  |  |  | Cold; resp |  |  |
|  | 7 | 4 | X | X | X | X | X | X | X |  |  | X | X | X |  |  | X |  |  | Bilateral ear infection, barking cough | Ear infection & Gastro |  |  |
|  | 10 | 5 |  |  |  |  |  |  |  |  | X |  |  |  |  |  |  |  |  | Pulling at ears | Bilateral ear infection* |  |  |
|  | 11 | 6 | X | X | X | X |  | X | X |  | X |  |  |  |  |  |  |  |  |  | Flu; resp* |  |  |
|  | 12 | 7 | X | X | X | X |  | X | X |  |  |  |  |  |  |  |  |  |  |  | Cold; resp |  |  |
|  | 16 | 8 | X | X | X | X |  | X |  |  | X | X | X |  |  |  |  |  |  |  | Gastro |  |  |
|  | 18 | 9 |  |  | X | X |  |  |  |  | X |  |  |  |  |  |  |  |  | Ear infection | Ear infection* |  |  |
|  | 18 | 10 | X |  | X | X |  | X |  |  |  |  |  |  |  |  |  |  |  |  | Cold; resp |  |  |
|  | 19 | 11 |  |  |  |  |  |  |  |  |  | X | X |  |  |  |  |  |  |  | Gastro* |  |  |
|  | 19 | 12 | X | X | X | X |  | X |  |  |  |  |  |  |  |  |  |  |  |  | Cold; resp |  |  |
|  | 19 | 13 |  |  |  |  |  |  |  |  |  | X | X |  |  |  |  |  |  |  | Gastro* |  |  |
|  | 19 | 14 | X |  | X | X | X | X |  |  | X |  |  |  |  |  |  |  |  |  | Cold/flu; resp |  |  |
|  | 20 | 15 | X |  | X | X | X | X |  |  | X |  |  |  |  |  | X |  |  | Fatigue, chills, shakes | Cold/flu; resp |  |  |
|  | 23 | 16 |  |  |  |  |  |  |  |  | X |  |  |  |  |  |  |  |  |  | Fever |  |  |
| Yes | 1 | 1 |  |  | X | X |  |  | X | X |  |  |  |  |  |  |  |  |  |  | Cold; resp | 11 | 10 |
|  | 2 | 2 | X | X | X | X | X |  | X |  |  |  |  |  |  |  |  |  |  | Stool is green | Cold; resp* |  |  |
|  | 5 | 3 | X |  | X | X | X |  |  |  |  |  |  |  |  |  | X |  |  |  | Cold; resp |  |  |
|  | 6 | 4 | X |  | X | X | X |  |  |  |  |  |  |  |  |  | X | X |  |  | Cold; resp |  |  |
|  | 7 | 5 | X |  |  | X |  |  |  |  |  |  |  |  |  |  | X |  |  |  | Cold; resp |  |  |
|  | 10 | 6 | X |  | X | X | X |  |  |  | X |  |  |  |  |  | X |  | X | Muscle pain | Flu; resp |  |  |
|  | 11 | 7 | X |  | X | X |  |  |  |  |  |  |  |  |  |  |  |  |  |  | Cold; resp |  |  |
|  | 14 | 8 |  |  |  |  |  |  |  |  | X |  |  |  |  |  |  |  |  |  | Fever |  |  |
|  | 12 | 9 | X |  |  |  |  |  |  |  | X |  |  |  |  |  |  |  |  | Clingy, reduced appetite | Cold/flu; resp |  |  |
|  | 12 | 10 |  |  |  | X |  |  |  |  | X |  |  |  |  |  |  |  |  | Clingy | Flu; resp |  |  |
|  | 13 | 11 | X |  |  | X |  |  |  |  |  |  |  |  |  |  | X |  |  | Drooling | Cold; resp |  |  |
|  | 14 | 12 | X |  |  | X |  |  |  |  |  |  |  |  |  |  |  |  |  |  | Cold; resp |  |  |
| Yes | 5 | 1 | X |  | X | X |  | X |  |  |  |  |  |  |  |  |  |  |  |  | Cold; resp | 3 | 7.33 |
|  | 9 | 2 |  |  |  |  |  |  |  |  | X |  |  | X |  |  |  |  |  | Reduced appetite & irritable | Flu; resp |  |  |
|  | 10 | 3 |  | X | X |  |  |  |  |  |  |  |  |  |  |  |  |  |  |  | Cold; resp |  |  |
| Yes | 1 | 1 | X | X | X | X | X | X | X | X |  |  |  |  |  |  |  |  |  |  | Cold; resp | 1 | 14 |
| Yes | 4 | 1 |  |  |  |  |  |  |  |  |  |  |  | X |  |  |  |  |  |  | Diaper rash & yeast infection* | 16 | 4.68 |
|  | 4 | 2 |  |  |  |  |  |  |  |  |  |  |  | X |  |  |  |  |  |  | Diaper rash & yeast infection* |  |  |
|  | 4 | 3 |  |  | X | X |  |  |  |  | X |  |  |  |  |  |  |  |  |  | Cold; resp |  |  |
|  | 9 | 4 |  |  | X | X |  | X |  |  |  |  |  |  |  |  |  |  |  |  | Cold; resp |  |  |
|  | 10 | 5 | X |  | X | X |  | X |  |  |  |  |  |  |  |  |  |  |  |  | Cold; resp |  |  |
|  | 12 | 6 |  |  |  | X |  | X |  |  |  |  |  |  |  |  |  |  |  |  | Cold; resp |  |  |
|  | 13 | 7 |  |  |  | X |  |  |  |  |  |  |  |  |  |  |  |  |  |  | Cold; resp |  |  |
|  | 13 | 8 |  |  |  |  |  |  |  |  |  |  | X | X |  |  |  |  |  |  | Gastro |  |  |
|  | 14 | 9 | X |  |  | X | X |  |  |  | X |  |  |  |  | X |  |  |  | lethargy | Cold; resp* |  |  |
|  | 16 | 10 |  |  | X | X |  |  |  |  |  |  |  |  |  |  |  |  |  |  | Cold; resp |  |  |
|  | 16 | 11 |  |  |  |  |  |  |  |  |  | X |  |  |  |  |  |  |  | Reduced appetite | Gastro |  |  |
|  | 16 | 12 | X |  |  | X |  |  |  |  |  |  |  |  |  |  |  |  |  |  | Cold; resp |  |  |
|  | 16 | 13 |  |  |  | X | X |  |  |  | X |  |  |  |  | X |  |  |  |  | Cold; resp |  |  |
|  | 16 | 14 | X |  |  | X |  |  |  |  |  |  |  |  |  |  |  |  |  |  | Cold; resp |  |  |
|  | 18 | 15 | X |  |  | X |  |  |  |  |  |  |  |  |  |  |  |  |  |  | Cold; resp |  |  |
|  | 19 | 16 | X |  |  |  |  |  |  |  |  |  |  |  |  |  |  |  |  |  | Cold; resp |  |  |
|  | 19 | 17 | X |  |  | X |  |  |  |  | X |  |  |  |  |  |  |  |  |  | Flu; resp |  |  |
|  | 20 | 18 | X |  |  |  |  |  |  |  | X |  |  |  |  |  |  |  |  | Ear ache | Bronchitis; resp* |  |  |
| Yes | 7 | 1 | X | X |  |  |  |  |  |  | X |  |  |  |  |  | X |  |  | Red throat | Cold; resp* | 4 | 6 |
|  | 11 | 2 | X | X | X | X | X |  |  |  | X | X |  |  |  |  |  |  |  |  | Cold; resp |  |  |
|  | 12 | 3 |  |  |  |  |  |  |  |  | X | X | X |  |  |  |  |  |  |  | Gastro |  |  |
|  | 20 | 4 |  |  |  |  |  |  |  |  |  | X | X |  | X |  |  |  |  |  | Gastro |  |  |
| Yes | 2 | 1 | X | X | X | X | X |  |  |  |  |  |  |  |  |  |  |  |  |  | Cold; resp | 5 | 4.6 |
|  | 5 | 2 |  |  | X | X | X |  |  |  |  |  |  |  |  |  |  |  |  |  | Cold; resp |  |  |
|  | 5 | 3 | X | X |  | X | X |  |  |  | X |  |  |  |  |  |  |  |  |  | Cold; resp |  |  |
|  | 6 | 4 |  | X |  | X | X |  |  |  |  |  |  |  |  |  |  |  |  |  | Cold; resp |  |  |
|  | 9 | 5 | X | X | X |  | X |  |  |  | X |  |  |  |  |  |  |  |  |  | Cold; resp |  |  |
| Yes | 7 | 1 |  |  |  |  |  |  |  |  |  | X |  | X |  |  |  |  |  |  | Reaction to vaccine | 1 | 1 |
|  | 10 | 2 |  |  | X | X |  |  |  |  |  |  |  |  |  |  |  |  |  |  | Cold; resp |  |  |
|  | 15 | 3 |  |  |  |  |  |  |  |  | X |  |  |  |  |  |  |  |  |  | Fever |  |  |
|  | 15 | 4 |  |  |  |  |  |  |  |  | X |  |  |  |  |  |  |  |  |  | Fever |  |  |
|  | 16 | 5 |  |  |  |  |  |  |  |  | X |  |  |  |  |  |  |  |  |  | Reaction to vaccine |  |  |
| Yes | 10 | 1 | X | X | X | X |  |  |  |  |  |  |  |  |  |  |  |  |  |  | Cold; resp | 2 | 10 |
|  | 12 | 2 | X |  | X | X | X |  | X |  |  |  | X |  |  |  | X |  |  | Irritable, sneezing | Cold; resp |  |  |
| Yes | 22 | 1 | X |  |  |  |  |  | X |  |  |  |  |  |  |  |  | X |  |  | Cold; resp | 4 | 3.5 |
|  | 21 | 2 |  |  |  |  |  |  |  |  |  | X |  |  |  |  |  |  |  |  | Gastro |  |  |
|  | 20 | 3 | X |  |  |  | X |  |  |  |  |  |  |  |  |  |  | X |  |  | Cold; resp |  |  |
|  | 19 | 4 |  |  | X |  |  |  |  |  |  |  |  |  |  |  |  | X |  |  | Cold; resp |  |  |
| Yes | 5 | 1 |  |  |  |  |  |  |  |  |  |  |  |  |  |  |  |  |  |  | Labial adhesion* |  |  |
|  | 7 | 2 | X | X | X | X | X | X |  | X | X |  |  | X |  | X |  |  |  |  | Cold; resp | 4 | 10.75 |
|  | 9 | 3 |  |  |  |  |  |  |  |  | X |  |  | X |  |  |  |  |  |  | Hand, Foot & Mouth Disease* |  |  |
|  | 11 | 4 | X | X | X | X | X | X |  |  |  |  |  |  |  |  | X |  |  | Eye discharge | Cold; resp |  |  |
|  | 17 | 5 |  |  |  |  |  |  |  |  |  | X | X | X |  |  |  |  |  |  | Gastro |  |  |
| Yes | 4 | 1 | X |  | X | X | X | X |  |  | X |  |  |  |  |  |  |  |  |  | Cold; resp | 8 | 12.1 |
|  | 4 | 2 | X |  | X | X | X | X |  |  |  |  |  |  |  |  |  |  |  |  | Cold; resp |  |  |
|  | 6 | 3 | X |  | X | X | X | X |  |  | X |  |  |  |  |  |  |  |  |  | Flu; resp |  |  |
|  | 7 | 4 | X | X | X | X | X | X | X |  | X |  |  |  |  |  |  |  |  |  | Flu; resp |  |  |
|  | 8 | 5 | X | X | X | X | X | X | X |  | X |  |  |  |  |  |  |  |  |  | Flu; resp |  |  |
|  | 9 | 6 | X | X | X | X |  | X |  |  | X |  |  |  |  |  |  |  |  |  | Flu; resp |  |  |
|  | 9 | 7 |  |  |  |  |  |  |  |  |  |  |  |  |  |  |  |  |  | White patches and sores in mouth | Thrush in mouth; Muc |  |  |
|  | 10 | 8 | X |  | X | X | X | X |  |  |  |  |  |  |  |  |  |  |  |  | Cold; resp |  |  |

*a medical professional was consulted; Mucosal (Muc); Respiratory (Resp); Gastrointestinal (Gastro)

Symptoms (1) Cough; (2) Phlegm; (3) Nasal congestion; (4) Nasal Secretion; (5) Running nose and eyes; (6) Cold; (7) Wheezing; (8) Difficulty breathing; (9) Fever; (10) Vomiting; (11) Diarrhea; (12) Rash; (13) Stomach pain; (14) Irritation and redness of eyes; (15) Sore throat; (16) Swollen glands in the neck; (17) Headache; Other- listed.

| **Table S3. Combination of subset models using variables within the global model** | | | | | | | | | | | | | |
| --- | --- | --- | --- | --- | --- | --- | --- | --- | --- | --- | --- | --- | --- |
| **combination#** | **(Intercept)** | **c.DHA** | **c.MOD** | **c.pets** | **c.preschool** | **c.probiotics** | **c.siblings** | **adj.Rsquare** | **df** | **logLik** | **AICc** | **delta** | **weight** |
| 17 | -4.20E-17 | NA | NA | NA | NA | 0.42 | NA | 0.15 | 3 | -17.05 | 41.10 | 0.00 | 0.11 |
| 49 | -9.41E-18 | NA | NA | NA | NA | 0.39 | 0.31 | 0.19 | 4 | -15.72 | 41.18 | 0.08 | 0.11 |
| 50 | 5.04E-18 | -0.28 | NA | NA | NA | 0.39 | 0.34 | 0.24 | 5 | -14.28 | 41.29 | 0.19 | 0.10 |
| 18 | -3.14E-17 | -0.25 | NA | NA | NA | 0.42 | NA | 0.18 | 4 | -15.98 | 41.71 | 0.60 | 0.08 |
| 25 | -6.35E-17 | NA | NA | NA | 0.15 | 0.41 | NA | 0.14 | 4 | -16.67 | 43.08 | 1.98 | 0.04 |
| 19 | -3.93E-17 | NA | 0.09 | NA | NA | 0.42 | NA | 0.12 | 4 | -16.94 | 43.61 | 2.51 | 0.03 |
| 21 | -5.15E-17 | NA | NA | 0.08 | NA | 0.42 | NA | 0.12 | 4 | -16.96 | 43.66 | 2.55 | 0.03 |
| 53 | -2.11E-17 | NA | NA | 0.10 | NA | 0.39 | 0.32 | 0.17 | 5 | -15.54 | 43.81 | 2.71 | 0.03 |
| 33 | -5.49E-18 | NA | NA | NA | NA | NA | 0.35 | 0.06 | 3 | -18.43 | 43.86 | 2.76 | 0.03 |
| 26 | -5.14E-17 | -0.24 | NA | NA | 0.13 | 0.41 | NA | 0.16 | 5 | -15.64 | 44.01 | 2.90 | 0.03 |
| 51 | -8.68E-18 | NA | 0.05 | NA | NA | 0.40 | 0.30 | 0.16 | 5 | -15.69 | 44.10 | 3.00 | 0.03 |
| 1 | -4.20E-17 | NA | NA | NA | NA | NA | NA | 0.00 | 2 | -19.81 | 44.11 | 3.00 | 0.03 |
| 57 | -1.02E-17 | NA | NA | NA | 0.00 | 0.39 | 0.31 | 0.16 | 5 | -15.72 | 44.17 | 3.07 | 0.02 |
| 34 | 9.10E-18 | -0.28 | NA | NA | NA | NA | 0.37 | 0.10 | 4 | -17.23 | 44.20 | 3.09 | 0.02 |
| 54 | -4.36E-18 | -0.27 | NA | 0.08 | NA | 0.39 | 0.34 | 0.22 | 6 | -14.16 | 44.32 | 3.22 | 0.02 |
| 20 | -2.91E-17 | -0.25 | 0.08 | NA | NA | 0.42 | NA | 0.15 | 5 | -15.88 | 44.50 | 3.39 | 0.02 |
| 52 | 5.48E-18 | -0.28 | 0.03 | NA | NA | 0.39 | 0.33 | 0.21 | 6 | -14.26 | 44.52 | 3.42 | 0.02 |
| 58 | 1.24E-17 | -0.28 | NA | NA | -0.03 | 0.39 | 0.36 | 0.21 | 6 | -14.26 | 44.53 | 3.43 | 0.02 |
| 22 | -3.84E-17 | -0.25 | NA | 0.05 | NA | 0.42 | NA | 0.15 | 5 | -15.93 | 44.59 | 3.49 | 0.02 |
| 2 | -3.14E-17 | -0.25 | NA | NA | NA | NA | NA | 0.02 | 3 | -18.94 | 44.88 | 3.78 | 0.02 |
| 29 | -8.07E-17 | NA | NA | 0.11 | 0.17 | 0.40 | NA | 0.11 | 5 | -16.47 | 45.67 | 4.56 | 0.01 |
| 27 | -6.19E-17 | NA | 0.11 | NA | 0.16 | 0.41 | NA | 0.11 | 5 | -16.49 | 45.71 | 4.61 | 0.01 |
| 9 | -6.78E-17 | NA | NA | NA | 0.18 | NA | NA | -0.01 | 3 | -19.36 | 45.72 | 4.62 | 0.01 |
| 37 | -1.75E-17 | NA | NA | 0.10 | NA | NA | 0.36 | 0.03 | 4 | -18.27 | 46.29 | 5.18 | 0.01 |
| 23 | -4.79E-17 | NA | 0.08 | 0.07 | NA | 0.42 | NA | 0.09 | 5 | -16.87 | 46.46 | 5.36 | 0.01 |
| 5 | -5.15E-17 | NA | NA | 0.08 | NA | NA | NA | -0.03 | 3 | -19.74 | 46.47 | 5.37 | 0.01 |
| 3 | -4.03E-17 | NA | 0.05 | NA | NA | NA | NA | -0.04 | 3 | -19.78 | 46.56 | 5.46 | 0.01 |
| 41 | -9.52E-18 | NA | NA | NA | 0.02 | NA | 0.34 | 0.02 | 4 | -18.43 | 46.59 | 5.49 | 0.01 |
| 35 | -5.34E-18 | NA | 0.01 | NA | NA | NA | 0.35 | 0.02 | 4 | -18.43 | 46.60 | 5.50 | 0.01 |
| 10 | -5.58E-17 | -0.24 | NA | NA | 0.16 | NA | NA | 0.01 | 4 | -18.53 | 46.79 | 5.69 | 0.01 |
| 28 | -5.02E-17 | -0.24 | 0.10 | NA | 0.15 | 0.41 | NA | 0.14 | 6 | -15.48 | 46.97 | 5.87 | 0.01 |
| 38 | -6.24E-19 | -0.27 | NA | 0.08 | NA | NA | 0.38 | 0.07 | 5 | -17.12 | 46.98 | 5.87 | 0.01 |
| 30 | -6.53E-17 | -0.24 | NA | 0.09 | 0.15 | 0.40 | NA | 0.14 | 6 | -15.51 | 47.02 | 5.92 | 0.01 |
| 55 | -2.00E-17 | NA | 0.03 | 0.10 | NA | 0.40 | 0.31 | 0.14 | 6 | -15.52 | 47.05 | 5.95 | 0.01 |
| 61 | -2.71E-17 | NA | NA | 0.10 | 0.03 | 0.39 | 0.30 | 0.14 | 6 | -15.53 | 47.07 | 5.96 | 0.01 |
| 42 | 1.32E-17 | -0.28 | NA | NA | -0.02 | NA | 0.39 | 0.06 | 5 | -17.22 | 47.18 | 6.07 | 0.01 |
| 36 | 9.04E-18 | -0.28 | 0.00 | NA | NA | NA | 0.38 | 0.06 | 5 | -17.23 | 47.18 | 6.08 | 0.01 |
| 59 | -1.22E-17 | NA | 0.05 | NA | 0.02 | 0.40 | 0.29 | 0.13 | 6 | -15.68 | 47.36 | 6.26 | 0.00 |
| 6 | -3.84E-17 | -0.25 | NA | 0.05 | NA | NA | NA | -0.01 | 4 | -18.90 | 47.54 | 6.44 | 0.00 |
| 4 | -3.01E-17 | -0.25 | 0.04 | NA | NA | NA | NA | -0.01 | 4 | -18.92 | 47.57 | 6.47 | 0.00 |
| 24 | -3.51E-17 | -0.25 | 0.07 | 0.05 | NA | 0.42 | NA | 0.12 | 6 | -15.85 | 47.70 | 6.60 | 0.00 |
| 56 | -3.70E-18 | -0.27 | 0.02 | 0.08 | NA | 0.39 | 0.34 | 0.18 | 7 | -14.15 | 47.91 | 6.80 | 0.00 |
| 62 | -3.39E-19 | -0.27 | NA | 0.08 | -0.02 | 0.39 | 0.35 | 0.18 | 7 | -14.16 | 47.92 | 6.81 | 0.00 |
| 13 | -8.60E-17 | NA | NA | 0.12 | 0.20 | NA | NA | -0.03 | 4 | -19.18 | 48.09 | 6.99 | 0.00 |
| 60 | 1.12E-17 | -0.28 | 0.03 | NA | -0.03 | 0.39 | 0.35 | 0.18 | 7 | -14.25 | 48.10 | 7.00 | 0.00 |
| 11 | -6.67E-17 | NA | 0.08 | NA | 0.18 | NA | NA | -0.04 | 4 | -19.28 | 48.31 | 7.20 | 0.00 |
| 31 | -7.78E-17 | NA | 0.10 | 0.10 | 0.18 | 0.41 | NA | 0.08 | 6 | -16.33 | 48.65 | 7.55 | 0.00 |
| 7 | -4.95E-17 | NA | 0.04 | 0.07 | NA | NA | NA | -0.07 | 4 | -19.71 | 49.17 | 8.06 | 0.00 |
| 45 | -2.73E-17 | NA | NA | 0.11 | 0.04 | NA | 0.33 | -0.01 | 5 | -18.25 | 49.23 | 8.13 | 0.00 |
| 39 | -1.77E-17 | NA | -0.01 | 0.10 | NA | NA | 0.36 | -0.01 | 5 | -18.27 | 49.27 | 8.17 | 0.00 |
| 14 | -7.07E-17 | -0.23 | NA | 0.09 | 0.18 | NA | NA | -0.02 | 5 | -18.41 | 49.54 | 8.44 | 0.00 |
| 43 | -1.00E-17 | NA | 0.01 | NA | 0.02 | NA | 0.33 | -0.02 | 5 | -18.42 | 49.57 | 8.47 | 0.00 |
| 12 | -5.50E-17 | -0.24 | 0.07 | NA | 0.17 | NA | NA | -0.02 | 5 | -18.47 | 49.66 | 8.56 | 0.00 |
| 40 | -1.11E-18 | -0.27 | -0.02 | 0.08 | NA | NA | 0.38 | 0.03 | 6 | -17.12 | 50.24 | 9.14 | 0.00 |
| 46 | -4.38E-19 | -0.27 | NA | 0.08 | 0.00 | NA | 0.38 | 0.03 | 6 | -17.12 | 50.25 | 9.15 | 0.00 |
| 32 | -6.27E-17 | -0.23 | 0.09 | 0.08 | 0.16 | 0.41 | NA | 0.11 | 7 | -15.38 | 50.36 | 9.26 | 0.00 |
| 44 | 1.36E-17 | -0.28 | -0.01 | NA | -0.02 | NA | 0.39 | 0.02 | 6 | -17.22 | 50.45 | 9.34 | 0.00 |
| 8 | -3.67E-17 | -0.25 | 0.04 | 0.05 | NA | NA | NA | -0.05 | 5 | -18.88 | 50.49 | 9.39 | 0.00 |
| 63 | -2.80E-17 | NA | 0.04 | 0.10 | 0.04 | 0.39 | 0.29 | 0.10 | 7 | -15.51 | 50.62 | 9.51 | 0.00 |
| 15 | -8.40E-17 | NA | 0.06 | 0.11 | 0.21 | NA | NA | -0.07 | 5 | -19.12 | 50.97 | 9.87 | 0.00 |
| 64 | -9.35E-19 | -0.27 | 0.02 | 0.07 | -0.01 | 0.39 | 0.35 | 0.14 | 8 | -14.15 | 51.88 | 10.78 | 0.00 |
| 47 | -2.73E-17 | NA | 0.00 | 0.11 | 0.04 | NA | 0.33 | -0.05 | 6 | -18.25 | 52.51 | 11.40 | 0.00 |
| 16 | -6.91E-17 | -0.23 | 0.06 | 0.09 | 0.19 | NA | NA | -0.06 | 6 | -18.36 | 52.72 | 11.62 | 0.00 |
| 48 | 6.65E-20 | -0.28 | -0.02 | 0.08 | -0.01 | NA | 0.39 | -0.01 | 7 | -17.12 | 53.84 | 12.74 | 0.00 |

Selected-models used in “avg-selected-models” based on delta AICc values <2

**Standardized coefficients** All-models used in “avg-all-models” selected based on cumulative sum of weight-scores <95
